# Inhibition of TRPA1, Endoplasmic Reticulum Stress, Human Airway Epithelial Cell Damage, and Ectopic *MUC5AC* Expression by Vasaka (*Adhatoda vasica*; Malabar Nut) Tea

**DOI:** 10.3390/ph16060890

**Published:** 2023-06-17

**Authors:** Tosifa A. Memon, Lili Sun, Marysol Almestica-Roberts, Cassandra E. Deering-Rice, Philip J. Moos, Christopher A. Reilly

**Affiliations:** 1 Department of Pharmacology and Toxicology, College of Pharmacy, University of Utah Health, Salt Lake City, UT 84112, USA; memon.tosifa@gmail.com (T.A.M.); lili.sun@pharm.utah.edu (L.S.); m.almestica@utah.edu (M.A.-R.); cassandra.rice@utah.edu (C.E.D.-R.); philip.moos@pharm.utah.edu (P.J.M.); 2 Center for Human Toxicology, College of Pharmacy, University of Utah Health, Salt Lake City, UT 84112, USA

**Keywords:** Vasaka, TRPA1, airway epithelium, mucus hypersecretion, *MUC5AC*, lung injury, wood smoke, biomass smoke

## Abstract

This study tested whether a medicinal plant, Vasaka, typically consumed as a tea to treat respiratory malaise, could protect airway epithelial cells (AECs) from wood smoke particle-induced damage and prevent pathological mucus production. Wood/biomass smoke is a pneumotoxic air pollutant. Mucus normally protects the airways, but excessive production can obstruct airflow and cause respiratory distress. Vasaka tea pre- and co-treatment dose-dependently inhibited mucin 5AC (*MUC5AC*) mRNA induction by AECs treated with wood smoke particles. This correlated with transient receptor potential ankyrin-1 (TRPA1) inhibition, an attenuation of endoplasmic reticulum (ER) stress, and AEC damage/death. Induction of mRNA for anterior gradient 2, an ER chaperone/disulfide isomerase required for *MUC5AC* production, and TRP vanilloid-3, a gene that suppresses ER stress and wood smoke particle-induced cell death, was also attenuated. Variable inhibition of TRPA1, ER stress, and *MUC5AC* mRNA induction was observed using selected chemicals identified in Vasaka tea including vasicine, vasicinone, apigenin, vitexin, isovitexin, isoorientin, 9-oxoODE, and 9,10-EpOME. Apigenin and 9,10-EpOME were the most cytoprotective and mucosuppressive. Cytochrome P450 1A1 (CYP1A1) mRNA was also induced by Vasaka tea and wood smoke particles. Inhibition of CYP1A1 enhanced ER stress and *MUC5AC* mRNA expression, suggesting a possible role in producing protective oxylipins in stressed cells. The results provide mechanistic insights and support for the purported benefits of Vasaka tea in treating lung inflammatory conditions, raising the possibility of further development as a preventative and/or restorative therapy.

## 1. Introduction

This study tested the hypothesis that tea prepared from the medicinal plant, Vasaka (*Adhatoda vasica*; Malabar Nut), could attenuate airway epithelial cell (AEC) damage and ectopic *MUC5AC* expression in an in vitro model of WSPM-induced AEC/lung injury. Furthermore, it was investigated whether specific chemicals in Vasaka could replicate the effects of the tea. Despite the purported benefits of Vasaka in treating respiratory conditions [1,2,3], the mechanisms via which Vasaka acts have not been elucidated. Additionally, potential synergistic and/or antagonistic effects among Vasaka components have not been reported. Thus, while Vasaka tea may be useful as a homeopathic therapy, it may be possible to further optimize the beneficial effects using Vasaka-derived compounds that target regulatory pathways controlling AEC injury and excessive *MUC5AC* expression.

Breathing polluted air increases risks of infection, asthma, and chronic obstructive pulmonary disease (COPD), among other effects [4,5,6,7,8]. AEC damage may contribute to air pollution-associated morbidities. Wood and biomass smoke particulate materials (WSPM) are pneumotoxic and have the ability to preferentially stimulate mucin 5AC (*MUC5AC*) expression by human AECs in vitro and in mice following oropharyngeal delivery of suspended WSPM through the activation of transient receptor potential ankyrin-1 (TRPA1) and endoplasmic reticulum (ER) stress [9,10], Additionally, AEC damage triggers activation of epidermal growth factor receptor (EGFR) to drive *MUC5AC* expression. Accordingly, EGFR ligands can promote *MUC5AC* expression [9].

The effect of WSPM on AECs and *MUC5AC* expression is of interest because of the frequency at which humans are exposed to WSPM, and both AEC damage and excessive mucus production are consequential to human respiratory health. Common sources of exposure to WSPM include wood/biomass-burning in stoves, fireplaces, campfires, and wildfires. Depending upon the exposure (dose and duration), WSPM can cause mild reversible to severe irreversible lung injury [11,12,13]. Short-term exposure has been linked to increased rates of hospital admissions for respiratory complications [12,14,15,16], while long-term exposure has been associated with chronic disease exacerbation and causation, including asthma, emphysema, COPD, and “hut lung” [17,18].

Airway mucus contains gel-forming glycoproteins known as mucins. *MUC5AC* and mucin 5B (MUC5B) are secreted mucins. Differential expression of *MUC5AC* and MUC5B has been shown to affect the viscoelasticity of airway mucus [19,20]. MUC5B has been shown to be preferentially expressed in the airways of healthy people and mice, and to play an essential role in trapping pathogens and pollutants entering the airway [21,22]. However, *MUC5AC* has been found to be overexpressed in COPD patients and mice primed with allergens, where it plays a role in obstruction and airway hyper-reactivity [23,24,25]. Accordingly, targeting pathways leading to WSPM-induced AEC/lung injury and *MUC5AC* production may be advantageous [26].

## 2. Results

### 2.1. Vasaka Tea Attenuated MUC5AC mRNA Induction by AECs

**Figure 1:**
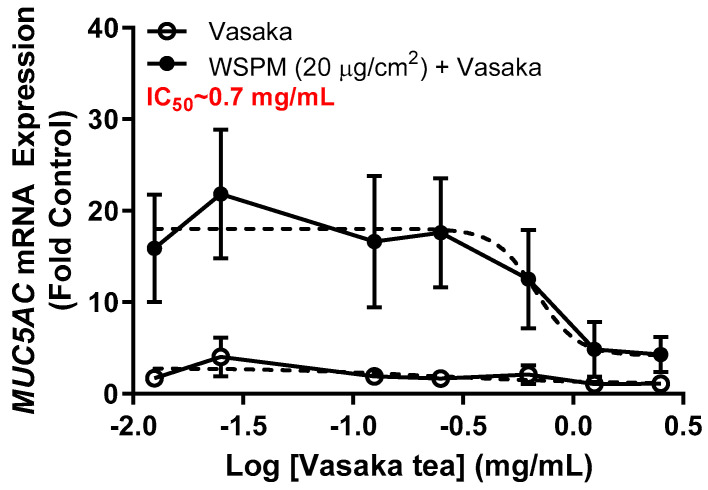
Concentration-dependent inhibition of *MUC5AC* mRNA expression by Vasaka tea. HBEC3-KT cells were pre- (2 h) and cotreated with Vasaka tea diluted in treatment medium with (solid circles) and without (open circles) WSPM (2.5 mg/mL) for 24 h (*n* = 3). *MUC5AC* mRNA expression was quantified using qPCR. Data were modeled using the log (antagonist) vs. response equation in GraphPad 9.5 software to predict the IC_50_ value.

Inhibition of WSPM-induced *MUC5AC* mRNA induction by Vasaka tea in HBEC3-KT AECs was evaluated. Vasaka tea was prepared, diluted in cell culture medium, and applied to cells for 2 h. After 2 h, fresh medium with varying percentages of Vasaka tea with and without WSPM (20 μg/cm^2^) was applied. The expression of *MUC5AC* mRNA was quantified 24 h later using qPCR (Figure 1). Note that we previously showed that *MUC5AC* mRNA induction is correlated with increased *MUC5AC* protein expression (periodic acid–Schiff staining and immunocytochemical analysis of cells) and secretion (enzyme-linked immunosorbent assay; ELISA) by AECs [9]. Here, Vasaka tea pre- and co-treatment at concentrations ≥ 0.625 mg/mL suppressed *MUC5AC* mRNA induction. The IC_50_ was approximately 0.7 mg/mL.

### 2.2. Vasaka Tea Attenuated TRPA1 Activation

**Figure 2:**
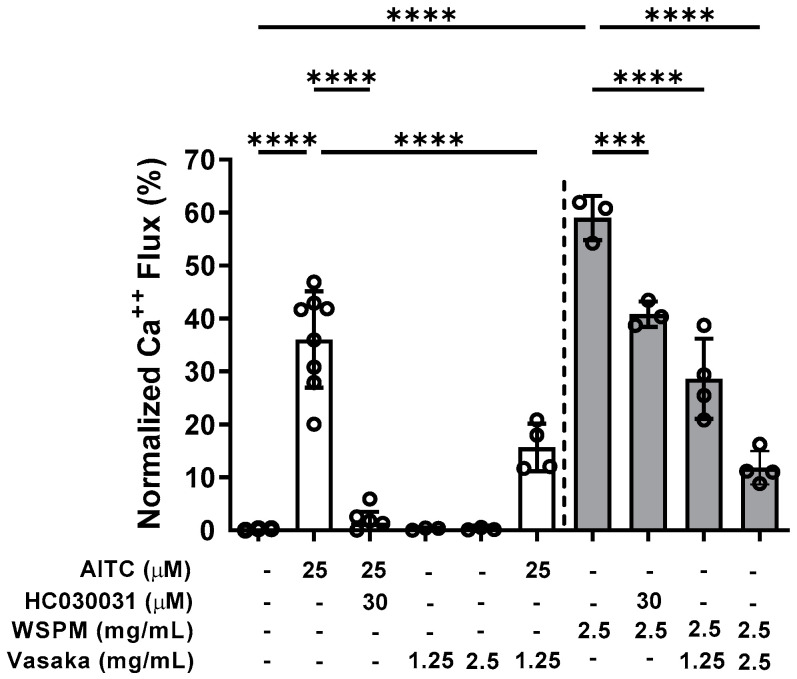
Inhibition of TRPA1-mediated calcium flux by Vasaka tea in HBEC3-KT cells treated with the TRPA1 agonist AITC (25 μM; white bars) or WSPM (2.5 mg/mL or 195 μg/cm^2^; gray bars). Replicates within each group are shown as open circles. HC-030031 is a TRPA1 antagonist. Vasaka tea was diluted in medium, and cells were pretreated with HC-030031 and Vasaka tea for 30 min prior to agonist (AITC or WSPM) application. Data were analyzed by one-way ANOVA with post hoc testing for significance among preselected groups using the Bonferroni test (*n* = 4–8). *** *p* < 0.001, **** *p* < 0.0001.

Inhibition of TRPA1 by Vasaka tea was subsequently tested. Pretreatment (>30 min), but not co-treatment of HBEC3-KT cells with Vasaka tea (1.25 and 2.5 mg/mL) decreased allyl-isothiocyanate (AITC)- (i.e., TRPA1-dependent) and WSPM-induced calcium flux akin to the TRPA1 antagonist HC-030031 (Figure 2).

### 2.3. Vasaka Tea Attenuated WSPM-Induced ER Stress, Oxidative Stress, and Other Cellular Pathways

**Figure 3:**
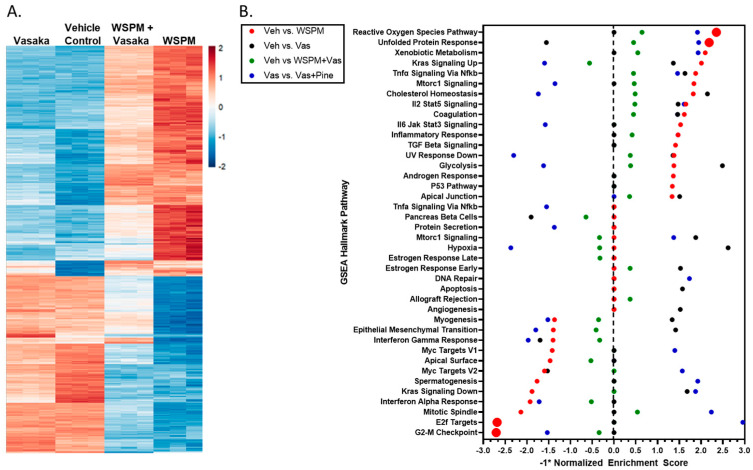
(**A**) Heatmap comparing the expression of the 1000 most changed transcripts in HBEC3-KT cells treated with WSPM compared to the vehicle control, as a function of treatment group. (**B**) Quantitative comparison of normalized enrichment score values multiplied by −1 (−1*NES) from pathway/GSEA of HBEC3-KT cells treated with vehicle (cell culture medium + 0.2% DMSO), WSPM (10 μg/cm^2^), Vasaka tea (1.25 mg/mL), or WSPM and Vasaka tea. Differential gene expression was determined using hciR and DESeq2 (1.30.1). Pathway analysis was performed using fgsea (1.18.0) using the Hallmarks Molecular Signatures Database. A negative value indicates enrichment in the comparison control group.

HBEC-3KT cells were treated with a vehicle control (0.2% dimethyl sulfoxide; DMSO in cell culture medium), WSPMP (10 μg/cm^2^), Vasaka tea (1.25 mg/mL), or a combination of both WSPM and Vasaka tea for 24 h. RNA sequencing was used to compare transcriptional changes associated with WSPM and Vasaka tea treatment. WSPM treatment induced robust changes to the AEC transcriptome, with many of the effects being attenuated by Vasaka tea cotreatment. Vasaka tea alone had few effects on the genes/pathways affected by WSPM. A comparison of the top 1000 genes exhibiting differential expression with WSPM treatment is shown in Figure 3A. The full dataset is publicly available.

Pathway analysis using Bioconductor Fast Geneset Enrichment Analysis (fgsea; or GSEA for short) was used to categorize cellular responses as pathways affected by WSPM and the Vasaka tea treatments. The hallmark *reactive oxygen species* (ROS) pathway (i.e., genes upregulated by ROS) was the most significant pathway associated with WSPM treatment (*p*-adjusted value = 0.0012; nominal enrichment score = 2.325; Figure 3B), according to the returned statistics using a default of 1000 permutations. Akin to prior observations [10], the hallmark *unfolded protein response* (UPR) pathway (i.e., genes upregulated during UPR/ER stress) was the second most activated pathway (*p*-adjusted value = 0.0012; nominal enrichment score = 2.173). WSPM treatment was also associated with a decrease in the hallmark *E2F target* pathway (i.e., genes encoding cell cycle-related targets of E2F transcription factors; *p*-adjusted value = 0.0012; nominal enrichment score = −2.694) and *G2M checkpoint* (genes involved in the G2/M checkpoint and progression through the cell cycle; *p*-adjusted value = 0.0012; nominal enrichment score = −2.716). These latter phenomena presumably developed from cell-cycle arrest driven by activation of the unfolded protein/ER stress response, as previously described for WSPM and the ER stress/the unfolded protein response pathway in general [10,27,28,29].

**Figure 4:**
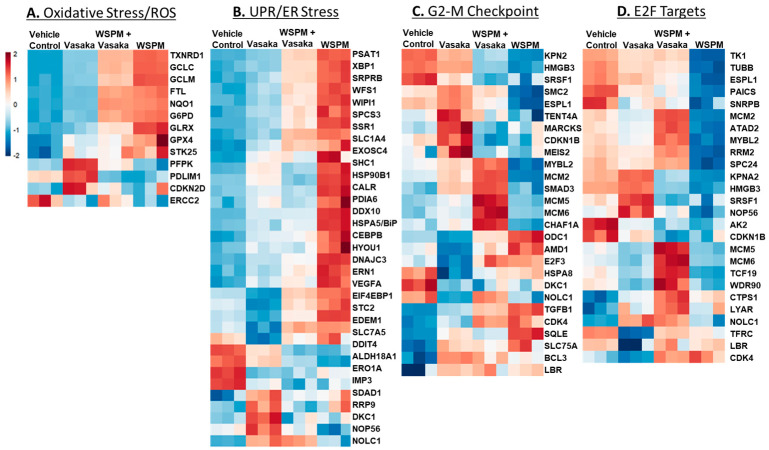
Heatmaps comparing the expression of significantly altered genes associated with the (**A**) hallmark *ROS/oxidative stress response* pathway, (**B**) the *unfolded protein response* pathway, (**C**) the hallmark *G2M checkpoint* pathway, and (**D**) the hallmark *E2F targets* pathway in HBEC3-KT cells treated with vehicle (cell culture medium + 0.2% DMSO), WSPM (10 μg/cm^2^), Vasaka tea (1.25 mg/mL), or WSPM and Vasaka tea.

The relative expression of specific genes driving the pathway predictions (i.e., leading edge genes; *p* < 0.05) is compared in Figure 4A–D. For example, for the ROS/oxidative stress pathway, thioredoxin reductase, genes involved in glutathione metabolism (GCLC glutamate–cysteine ligase catalytic subunit; GCLC, GCLM glutamate-cysteine ligase modifier subunit; GCLM), and glutaredoxin (GLRX) were upregulated by WSPM and attenuated by Vasaka tea cotreatment. Similarly, X-box-binding protein 1 (XBP1), protein disulfide isomerase family A member 6 (PDIA6), heat-shock protein family A member 5 (HSPA5/BiP/GRP78), and stanniocalcin 2 (STC2), among other genes commonly associated with the unfolded protein/ER stress pathway, were upregulated by WSPM treatment, and attenuated with Vasaka tea cotreatment. Other pathways of interest included hallmark *hypoxia*, *xenobiotic metabolism*, *Kras signaling (up and down)*, *glycolysis*, *cholesterol metabolism*, and *mitotic spindle* (leading edge analyses data are shown in Figure S1A–E). Regarding glycolysis, a related metabolomics analysis of BEAS-2B AECs treated with WSPM identified a time-dependent shift in metabolism in treated cells, wherein glycolytic metabolism was affected (i.e., the Warburg effect was observed; Figure S2).

**Figure 5:**
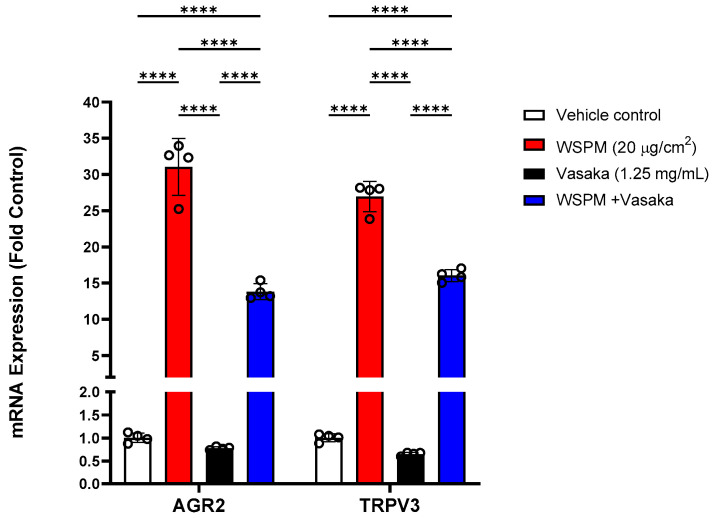
Comparison of *AGR2* and *TRPV3* mRNA expression in HBEC3-KT cells treated with vehicle (cell culture medium + 0.2% DMSO), WSPM (10 μg/cm^2^), Vasaka tea (1.25 mg/mL), or WSPM and Vasaka tea using qPCR. Replicates within each group are shown as open circles. **** *p* < 0.0001 using 2-way ANOVA and Tukey post-test to compare the effects of treatment on mRNA expression for each gene (*n* = 4).

Additional significantly modified genes induced by WSPM that were not linked with any of the pathways identified by GSEA included anterior gradient protein 2 homolog (AGR2) and transient receptor potential vanilloid-3 (TRPV3). AGR2 is an ER-associated protein disulfide isomerase necessary for *MUC5AC* expression and secretion [30], while TRPV3 induction by WSPM and other causes of cell injury including prototypical ER stress-inducing agents has previously been found to be protective [10,31]; induction of both genes was inhibited by Vasaka tea cotreatment (Figure 5).

### 2.4. Vasaka Tea Attenuated WSPM-Induced AEC Damage In Vitro

**Figure 6:**
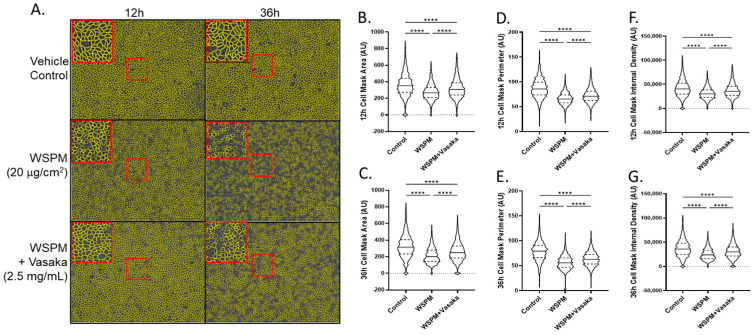
Comparison of HBEC3-KT morphological features and monolayer integrity as a function of WSPM and Vasaka tea treatments and time. Confluent monolayers of HBEC3-KT cells were treated with WSPM (20 μg/cm^2^) or WSPM + Vasaka tea (2.5 mg/mL) and imaged using an Incucyte live cell imaging system. (**A**) Representative images 12 h and 36 h post treatment with a cell mask overlaid in yellow. Red boxes indicate expanded regions of the image. Quantification of (**B**,**C**) cell area, (**D**,**E**) cell perimeter, and (**F**,**G**) internal density of cells at 12 and 36 h. **** *p* < 0.0001 using ANOVA and Tukey post-test to compare the average feature values as a function of treatments. Movies showing changes associated with the treatments are shown in the Supplementary Materials.

Vasaka tea cotreatment of HBEC-3KT cells also protected AECs from WSPM damage (Figure 6). HBEC3-KT cells treated with WSPM (20 μg/cm^2^) exhibited time-dependent disruption of cell morphology and monolayer integrity beginning within ~4 h of treatment, with evidence of mounting damage occurring over ~24 h (Figure S3 Movies). AEC damage was characterized by a decrease in mean area, perimeter, and internal density. While the AEC monolayer remained distinguishable from control cells when cotreated with WSPM and Vasaka tea, protection was observed as indicated by the partial attenuation of WSPM-induced changes in cell morphology and monolayer integrity.

### 2.5. Vasaka Components Exhibited Variable Inhibition of TRPA1, ER and Oxidative Stress, and MUC5AC Induction

**Figure 7:**
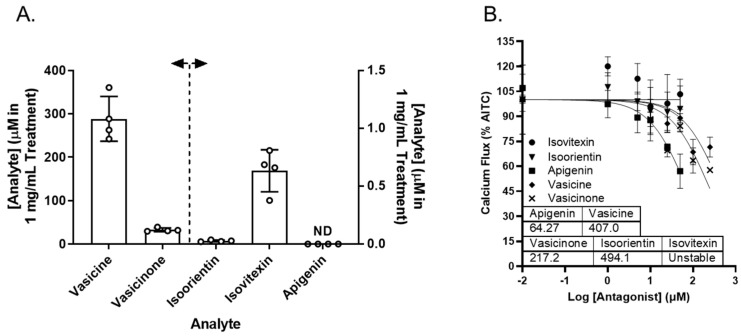
(**A**) Estimated concentrations of vasicine, vasicinone, isoorientin, isovitexin, and apigenin in a 1 mg/mL solution of Vasaka tea. Analytes were quantified by LC/MS/MS (*n* = 4 extracts). The dashed line and arrows indicate which y-axis the data are plotted on and replicates within each group are shown as open circles. A representative LC/MS/MS chromatogram is shown in Figure S4. (**B**) Inhibition of AITC-induced (TRPA1-dependent) calcium flux in HBEC3-KT cells treated with increasing concentrations of apigenin, vasicine, and vasicinone and stimulated with AITC (25 μM). Data were modeled using the log (antagonist) vs. response equation in GraphPad 9.5 software to estimate the IC_50_ value (*n* = 3–4).

Vasaka tea contains numerous chemicals that may or may not inhibit TRPA1, prevent ER and/or oxidative stress, or attenuate *MUC5AC* induction. Reported chemicals include vasicine, vasicinone, apigenin, and a variety of glycated flavonoids [32]. Vasicine, vasicinone, isoorientin, isovitexin, and apigenin were quantified in the Vasaka tea using liquid chromatography–tandem mass spectrometry (LC/MS/MS). The concentrations estimated for a 1 mg/mL solution of Vasaka tea were as follows (in μM): vasicinone (290 ± 52), vasicine (33 ± 4), isoorientin (0.27 ± 0.007), isovitexin (0.6 ± 0.2), and apigenin (<LLOQ; Figure 7A and Figure S4); vitexin is apigenin-8-C-glucoside, isovitexin is apigenin-6-C-glucoside, and isoorientin is luteolin-6-C-glucoside. The structures of these compounds and mirror match spectra from the untargeted LC/MS/MS analyses described below are shown in Figure S4B. While free apigenin was not detected in the Vasaka tea, it may be liberated from C- and O-linked glucosides (e.g., schaftoside, isoschaftoside, vitexin, isovitexin, vicinin, and apiin) [33,34,35,36,37], and it has been reported to occur in Vasaka extracts by others [32]. Accordingly, apigenin was included in further studies.

Vasicine, vasicinone, isoorientin, isovitexin, and apigenin were evaluated as TRPA1 antagonists. Vasicine, vasicinone, and apigenin dose-dependently inhibited TRPA1, albeit with variable and relatively low potency. Isovitexin and isoorientin had minimal inhibitory effect up to 50 μM. The IC_50_ values for vasicine, vasicinone, isoorientin, and apigenin were estimated to be ~407, 217, 494, and 64 μM, respectively (Figure 7B).

**Figure 8:**
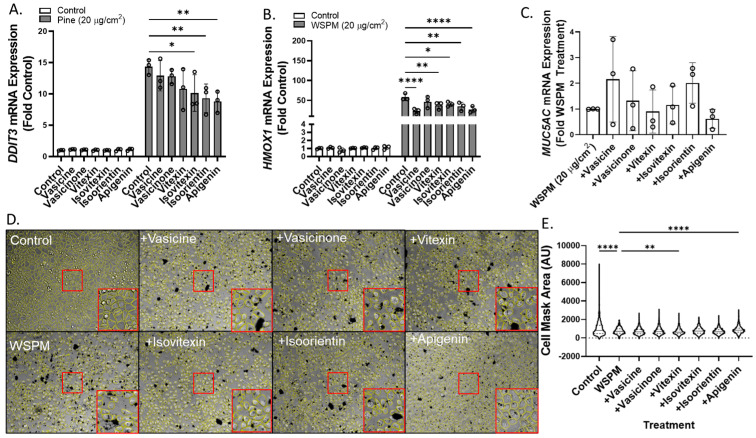
Comparison of (**A**) *DDIT3*, (**B**) *HMOX1*, and (**C**) *MUC5AC* mRNA expression in HBEC3-KT cells treated with vehicle (cell culture medium + 0.2% DMSO), WSPM (20 μg/cm^2^), and various concentrations of Vasaka tea components using qPCR (vasicine = 300 μM; vasicinone = 50 μM; vitexin = 2.5 μM; isovitexin = 2.5 μM; isoorientin = 2.5 μM; apigenin = 2.5 μM, based on Figure 7A. replicates within each group are shown as open circles. * *p* < 0.05, ** *p* < 0.01 and **** *p* < 0.0001 using two-way ANOVA and Dunnett post-test to compare the effects of treatment on mRNA expression for each gene compared to the control (*n* = 3). (**D**) Representative images 24 h post treatment collected using a Molecular Devices Image Express PICO with cell masks overlaid in yellow. Red boxes indicate expanded regions of the image. (**E**) Cell area as a function of treatments. ** *p* < 0.01 and **** *p* < 0.0001 using ANOVA and Dunnett post-test to compare to the effects of WSPM treatment.

Vasicine, vasicinone, isoorientin, isovitexin, and apigenin were also tested for the ability to attenuate ER stress (DNA damage-inducible transcript-3; *DDIT3*), oxidative stress (heme oxygenase-1; *HMOX1*), and *MUC5AC* mRNA induction using concentrations predicted to be in Vasaka tea when applied to cells at a concentration of ~1–2 mg/mL (Figure 8A–C). Vasicine (300 μM), vasicinone (50 μM), and vitexin (2.5 μM) failed to inhibit *DDIT3* induction, but isovitexin, isoorientin, and apigenin (all at 2.5 μM) reduced this response ~30%. All compounds also slightly reduced *HMOX1* induction, but only apigenin (2.5 μM) appeared to reduce *MUC5AC* mRNA induction. Lastly, apigenin and vitexin, but not isoorientin, vasicine, vasicinone, or isovitexin exhibited protective effects with respect to AEC monolayer damage (Figure 8D,E).

### 2.6. Vasaka Tea Is a Complex Mixture

Vasaka tea was also analyzed using untargeted LC/MS/MS and the Global Natural Products Social Molecular Networking (GNPS) Knowledge Base library search function [38] to identify additional Vasaka tea components (i.e., dereplication). Representative positive and negative electrospray ionization (+ and − ESI) LC/MS chromatograms are shown in Figure S5A,B. The GNPS results summary file is also included in the Supplementary Materials, and mzML raw data files are available upon request. To summarize, multiple previously reported chemicals were identified, including vasicine, vasicinone, isovitexin, vitexin, and numerous other flavonoids and glycated flavonoids.

**Figure 9:**
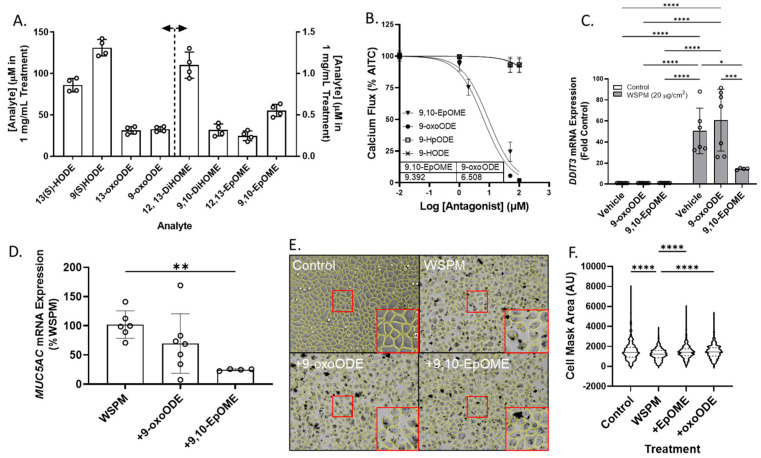
(**A**) Estimated concentrations of 13(S)- and 9(S)-HODE, 13-oxo and 9-oxoODE, 12,13-and 9,10-DiHOME, and 12,13- and 9,10-EpOME in a 1 mg/mL solution of Vasaka tea. Analytes were quantified by LC/MS/MS (*n* = 4 extracts). The dashed line and arrows indicate which y-axis the data are plotted on and replicates within each group are shown as open circles. A representative LC/MS/MS chromatogram is shown in Figure S6F. (**B**) Inhibition of AITC-induced (TRPA1-dependent) calcium flux in HBEC3-KT cells treated with various concentrations of oxylipins and stimulated with AITC (25 μM). Data were modeled with the log (antagonist) vs. response equation in GraphPad 9.5 software to estimate the IC_50_ value (*n* = 3–6). (**C**) *DDIT3* and (**D**) *MUC5AC* mRNA expression in HBEC3-KT cells treated with WSPM (20 μg/cm^2^) with and without pre- (30 min) and cotreatment with 9-oxoODE or 9,10-EpOME (1 μM). * *p* < 0.05, ** *p* < 0.01, *** *p* < 0.001 and **** *p* < 0.0001 using two-way ANOVA and a Tukey post hoc test comparing all groups (panel **D**) and ANOVA with a Dunnett post-test comparing the effects of treatment relative to WSPM treatment (panel **C**). *n* = 4–7. (**E**) Representative images 24 h post treatment collected using a Molecular Devices Image Express PICO with cell masks overlaid in yellow. Red boxes indicate expanded regions of the image. (**F**) Cell area as a function of treatments. **** *p* < 0.0001 using ANOVA and Dunnett post-test to compare to the effects of WSPM treatment. Data showing TRPA1 agonist and antagonist effects of 9-oxoODE, 9-HpODE, 9,10-EpOME, and 9-HODE at 50 μM are shown in Figure S7.

Previously unreported linoleic acid-derived oxylipins were also identified in Vasaka tea, including 9,10- and 12,13-EpOME (epoxy-9Z-octadecenoic acids), 9,10- and 12,13-DiHOME (dihydroxyoctadec-9-enoic acids), 9- and 13-HODE (hydroxyoctadecadienoic acids), 9-oxoODE (9-oxo-10E,12Z-octadecadienoic acid), and 9- and 13-HpODE (hydroperoxy-9Z,11E-octadecadienoic acids). These results were verified using water to prepare Vasaka tea as opposed to cell culture medium which contained linoleic acid. Mirror match spectra of these compounds as determined by GNPS analysis are shown in Figure S6A–E, and a chromatogram from targeted oxylipin analysis is shown in Figure S6F. Quantification of linoleic acid-derived oxylipins in Vasaka tea prepared in water by LC/MS/MS estimated concentrations in a 1 mg/mL Vasaka tea solution to be as follows (in mM): 12,13-DiHOME (1.1 ± 0.2), 9,10-DiHOME (0.32 ± 0.07), 13(S)-HODE (86 ± 8), 9(S)-HODE (131 ± 9), 13-oxoODE (31 ± 4), 9-oxoODE (33 ± 3), 12,13-EpOME (0.25 ± 0.06), and 9,10-EpOME (0.55 ± 0.07) (Figure 9A).

Both 9,10-EpOME and 9-oxoODE were found to inhibit TRPA1 activation by AITC (Figure 9B; IC_50_ ~9.4 and 6.5 μM, respectively), but only 9,10-EpOME reduced *DDIT3* and *MUC5AC* mRNA induction by WSPM (Figure 9C,D). 9,10-EpOME and, to a lesser extent, 9-oxoODE were also cytoprotective (Figure 9E,F). Of note, 9-HpODE was a TRPA1 agonist according to the inhibition of elicited calcium flux by the TRPA1 antagonist A967079, while 9-HODE had minimal effect on TRPA1 activity (Figure S7).

### 2.7. CYP1A1 Limited WSPM Toxicity

**Figure 10:**
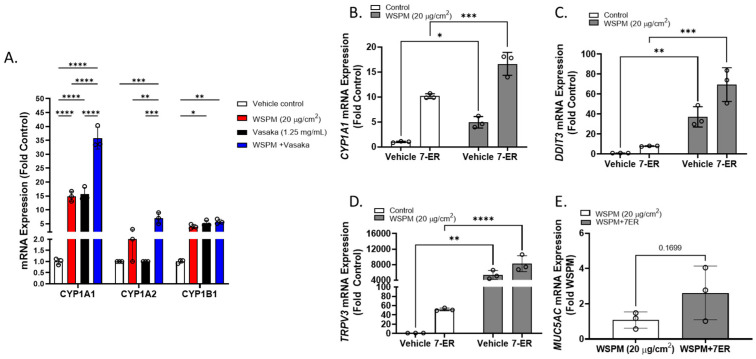
(**A**) Comparison of *CYP1A1*, *1A2*, and *1B1* mRNA expression by HBEC3-KT cells treated with vehicle (cell culture medium + 0.2% DMSO), WSPM (10 μg/cm^2^), Vasaka tea (1.25 mg/mL), or WSPM and Vasaka tea. Replicates within each group are shown as open circles. Data were analyzed using two-way ANOVA with a Tukey post-test comparing the effects of treatments (*n* = 3). * *p* < 0.05, ** *p* < 0.01, *** *p* < 0.001, **** *p* < 0.0001. Expression of mRNA for (**B**) *CYP1A1*, (**C**) *DDIT3*, (**D**) *TRPV3*, and (**E**) *MUC5AC* as a function of WSPM treatment with and without pre- (2 h) and cotreatment with the CYP1A1 inhibitor 7-ethoxyresorufin (7-ER) (*n* = 3). Data were analyzed using two-way ANOVA and a Bonferroni post-test to compare the effects of each treatment. * *p* < 0.05, ** *p* < 0.01, *** *p* < 0.001, **** *p* < 0.0001.

Cytochrome P450 1A1 (CYP1A1) was among the top dysregulated genes in AECs treated with WSPM and Vasaka tea (Figure 10A and Figure S1B). Pre- and cotreatment of HBEC3-KT cells with the CYP1A1 inhibitor 7-ethoxyresorufin (7-ER) [39,40] enhanced *CYP1A1* mRNA induction by WSPM akin to Vasaka tea (Figure 10B), enhanced the induction of *DDIT3* and *TRPV3* (ER stress markers; Figure 10C,D), and promoted *MUC5AC* mRNA expression (Figure 10E), suggesting that inhibition of CYP1A1 by Vasaka tea (likely by flavonoids) [41,42] may underlie the superinduction phenomenon, and that oxylipins produced by CYP1A1 (e.g., 9,10-EpOME) [43] may normally restrict injury and *MUC5AC* induction by WSPM.

## 3. Discussion

WSPM can be a prevalent household and ambient pollutant, and, in recent years, wildfires in North America have set records for the number of acres burned. While the type of biomass burned affects the characteristics and seemingly the relative potency of the resulting emissions as a pneumotoxin, we found that particle concentrates derived from burning pine, mesquite, sage, apple, range grass, sheep and cow dung, juniper, and other materials share the capacity to activate TRPA1, trigger ER stress, and induce *MUC5AC* expression [9,10]. While the selectivity for *MUC5AC* induction can also vary by WSPM and pollutant source/type, we and others have also found that TRPA1 can be activated and ER stress initiated by a variety of pneumotoxic ambient pollutants including diesel exhaust particles, cigarette smoke condensate, E-cigarette vaping products, and many other substances [9,10,44,45,46,47,48]. Thus, it is possible that the findings here could apply to a range of pneumotoxicants that humans can be exposed to on a frequent basis.

Currently, there are few if any therapeutics that prevent and/or directly treat lung injury, and available mucus-directed therapies are generally limited to expectorants and mucokinetics, although, in severe cases (e.g., ARDS), liquid ventilation can be used [49,50]. The most widely used mucus treatments include guaifenesin and anti-inflammatory oral and inhaled corticosteroids. Guaifenesin, derived from the Guaiac tree (FDA-approved and currently marketed as, e.g., Mucinex) acts as an expectorant by promoting discharge of mucus from the airways and facilitating mucociliary clearance. Guaifenesin is also weakly *MUC5AC*-suppressive [51] and can be paired with β_2_-agonists to aid in mucus clearance. Glucocorticoids, β_2_-agonists, and anticholinergic bronchodilators also relieve congestion and excess mucus production, and they are mainstay therapeutics for asthma [49]. While long-term therapy is possible with these agents, they are not known to prevent AEC injury as an origin for ectopic *MUC5AC* production. Furthermore, these agents are not guaranteed to work in all patients (e.g., people with glucocorticoid resistance), are costly, and have side-effects that may preclude their use in some circumstances and in some individuals. Chronic β_2_-agonist therapy specifically may be counterproductive in terms of *MUC5AC* suppression [52,53,54,55,56,57]. Thus, a problem with current therapies is that, while they aid in inflammation and mucus clearance to provide relief, cell damage and excess mucus production can continue, adversely affecting lung functions because AEC injury is not addressed [49,58]. Regarding the management of environmentally induced pulmonary effects on the respiratory tract, addressing acute and recurrent AEC injury and ectopic *MUC5AC* expression and hypersecretion may be key.

Vasaka (generally as a tea) is used in traditional medicine to treat asthma and tuberculosis due to its purported antitussive and mucolytic properties [1,2,59]. Bromhexine and its metabolite ambroxol are over-the-counter medicines available in some locations around the world, used to treat respiratory ailments involving excess mucus [2,3,60,61,62,63,64,65,66]. These synthetic molecules were inspired by vasicine and vasicinone, two of the most abundant chemicals in Vasaka, and they act by disrupting the structure of mucopolysaccharide fibers in sputum and decreasing viscosity such that the mucus can be cleared. Ambroxol has also been shown to stimulate surfactant production by alveolar type II cells, as well as inhibit immunoglobulin E (IgE)-dependent histamine release and the production of interleukin (IL)-4 and -13 by mast cells and basophils, in part, by modulating kinase signaling. Bromhexine, ambroxol, and another vasicine-based synthetic agent R8 have also been shown to be mildly *MUC5AC*-suppressive [51,67,68], but how these agents affect *MUC5AC* and AECs in general is not completely understood. Additionally, they are not widely available.

This study shows that Vasaka tea itself may be a source of therapeutic mucosuppressing agents that can also protect AECs from the damaging effects of WSPM, and possibly other TRPA1 agonists. Use of Vasaka in traditional medicine typically involves consumption as tea. If reports on the benefits of Vasaka on respiratory illnesses are true, it may be possible that one or more of the compounds in the tea have the capacity to distribute to the lungs through systemic circulation where they may limit AEC damage and/or associated proinflammatory effects triggered by the inciting stimulus. According to the results herein, this may involve TRPA1 inhibition, which, in the case of environmental exposures to pollutants that activate TRPA1 (WSPM, diesel exhaust, cigarette smoke, vaping chemicals, etc.), could be beneficial. Additional benefits may arise from antioxidants in the tea, flavonoids that modulate CYP enzyme functions and other cellular processes, bioactive lipids, and EGFR modulators. For example, apigenin, identified as protective in this study via apparent interactions with TRPA1, has also been reported to inhibit EGFR activation [69], which would become active downstream of TRPA1 in the scenario tested here. It is also possible that Vasaka tea could act at the systemic level to impact respiratory symptomatology. While not directly tested, modulation of TRPA1 in immune or other cell types (e.g., vascular endothelial cells in the alveolar capillaries) could be part of this process. Regardless, the abundance of bioactive flavonoids and oxylipins in Vasaka tea may contribute to the overall pneumoprotective and mucosuppressive effects of Vasaka tea.

Regarding potential therapeutic opportunities based on Vasaka and use of Vasaka itself, several protective and mucosuppressing compounds were identified in the tea that could potentially be delivered systemically or directly to the lungs on an as-needed basis. These include selected flavonoids (e.g., apigenin) and 9,10-EpOME. The finding that some oxylipins were TRPA1 antagonists was surprising as others have reported that they activate rodent TRPA1 [70], and we previously found that arachidonic acid-derived oxylipins produced from *Cyp1b1* metabolism could sensitize TRPA1 and promote chronic pain in mice [48]. While not shown, both 9,10-EpOME and 9-oxoODE were also able to activate TRPA1 at concentrations ~100 μM. Accordingly, careful consideration of dose would be needed to use these substances as therapeutics. Regardless, the data suggest a novel interaction between CYP enzymes and TRPA1 involving lipid-derived epoxides, wherein pre-emptive induction of CYPs by Vasaka (perhaps due to the presence of CYP1A1-inhibiting flavonoids) [71,72] may promote a net anti-inflammatory environment wherein TRPA1, ER stress, and *MUC5AC* induction are suppressed. This is consistent with the broad anti-inflammatory effects of other CYP1A inducers such as the endogenous aryl-hydrocarbon receptor ligand 6-formylindolo[3,2-b]carbazole (FICZ) [73]. Accordingly, dietary supplementation using, for example, linoleic acid, flavonoids, or perhaps even standardized extracts of Vasaka may be advantageous. To conclude, these ideas will require further study to establish feasibility, efficacy, and safety. However, the cumulative results from this study support efforts to develop Vasaka for preventing and treating respiratory conditions arising from exposure to toxic air pollutants that target the TRPA1 receptor.

## 4. Materials and Methods

### 4.1. Chemicals and Reagents

Vasicine, vasicinone, isoorientin, isovitexin, vitexin, apigenin, 12,13-DiHOME, 9,10-DiHOME, 13(S)-HODE, 9(S)-HODE, 13-oxoODE, 9-oxoODE, 12,13-EpOME and 9,10-EpOME, 9-HpODE, and 7-ethoxyresorufin were purchased from Cayman Chemical (Ann Arbor, MI, USA). AITC, ionomycin calcium salt, HC-030039, and A967079 were from Sigma-Aldrich (St. Louis, MO, USA). DMSO was from Fisher Scientific (Waltham, MA, USA).

### 4.2. WSPM

Preparation of pine WSPM was described in [47]. Briefly, ~10 g of 1 inch toothpick- to pencil-sized pieces of Austrian pine (from a tree growing in the Salt Lake Valley) were burned in a pipe furnace at 750 °C with constant air flow. WSPM was collected using an Anderson cascade impactor operated at 1 L/min, and fractions 6 and 7 (0.65–1.1 μm and 0.43–0.65 μm) were used. For experiments, WSPM concentrate was suspended in DMSO at ~100× the target concentration and diluted in cell culture medium for treatments.

### 4.3. Preparation of Vasaka Tea

Vasaka tea was prepared by incubating a suspension of organic Vasaka powder (Banyan Botanicals; 20 mg/mL) in cell culture medium for 30 min at 37 °C. The tube was vortexed every 5 min, and after 30 min the suspension was centrifuged at 20,000× *g* for 2 min, followed by filtration of the supernatant using a 0.22 μm syringe filter. The Vasaka tea was used fresh by diluting into cell culture medium or stored frozen as a concentrate at −80 °C for up to 1 month. Blank medium processed in parallel was used as a negative control.

### 4.4. Cell Culture

BEAS-2B (used only in a metabolomics study) and HBEC3-KT cells were purchased from ATCC (Rockville, MD, USA). Cells were maintained in a humidified cell culture incubator at 37 °C with a 95% air:5% CO_2_ atmosphere and sub-cultured every 2–3 days when cells reached ~80% confluence using trypsin. BEAS-2B cells were grown in LHC-9 medium (LHC-8 supplemented with 33 nM retinoic acid and 2.75 μM epinephrine), while HBEC3-KT cells were cultured in airway epithelial cell basal medium supplemented with a bronchial epithelial cell growth kit, 30 µg/mL geneticin, and 250 ng/mL puromycin.

### 4.5. Gene Expression Analysis/RNA qPCR

Cells were plated and treated at ~90% confluence. After treatment, total RNA was isolated using the Invitrogen PureLink RNA Mini Kit and quantified by UV absorbance on a Nanodrop One^c^. cDNA (2 μg) was synthesized using the Applied Biosystems High-Capacity cDNA Synthesis Kit with RNase inhibitor. Transcript abundance was quantified using TaqMan gene expression master mix (ThermoFisher, Waltham, MA, USA) on a Life Technologies QuantStudio 6 Flex instrument. The following TaqMan probe-based assays were used: human *MUC5AC* (Hs_01365616_m1); human HMOX1 (Hs01110250_m1); human DDIT3 (Hs_00358796_g1); human TRPV3 (Hs00376854_m1). The PCR programs used were according to supplier recommendations. Expression values were normalized to human β2-microglobulin (β*2M*; Hs00984230_m1), and to the average of the control samples.

### 4.6. TRPA1 Activity Assays

Calcium flux was measured using the Fluo-4 Direct assay kit and imaging on an EVOS FL Auto microscope at 10× magnification using a green fluorescent protein (GFP) filter as described [10,45,46,74]. Cells were plated in a flat bottom 96-well plate at ~25,000 cells/cm^2^ and assayed at 80–90%. Prior to assay, cells were loaded with Fluo-4 diluted 1:1 in calcium assay buffer (Hank’s balanced salt solution, with CaCl_2_ and MgCl_2_, buffered to pH 7.3 with 20 mM HEPES; ThermoFisher) at ~23 °C for 1 h in the dark. After 1 h, the Fluo-4 was removed, and the cells were washed with calcium assay buffer containing 1 mM probenecid and 0.75 mM trypan red (ATT Bioquest; Sunnyvale, CA, USA). Cells were pretreated for 30 min or 2 h with various agents by diluting in the wash buffer and in the desired treatment solutions (i.e., AITC or WSPM). Assays were performed using an EVOS FL auto microscope with an on-stage environmental chamber maintained at 37 °C with a 95% air/5% CO_2_ atmosphere. Agonist treatments (AITC or WSPM) were added to cells at 3× the desired final concentration in calcium assay buffer. Images were captured every 6 s for 72 s. Changes in fluorescence were quantified using a custom MATLAB program as previously described [46,74]. Reported values are from the 60 s timepoint and were corrected by subtracting the fluorescence response to a blank medium control (i.e., no agonist), then normalized to the response value at 72 s following ionomycin (20 μM) treatment applied after the 60 s image was taken.

### 4.7. Gene Expression Analysis/RNA Sequencing

RNA sequencing was performed at the High-Throughput Genomics Core Facility at the Huntsman Cancer Institute, University of Utah. Total RNA was extracted using the RNeasy Mini kit (Qiagen, Hilden, Germany) with on-column DNase digestion. RNA quality was assessed by Agilent TapeStation, and library construction was performed using the Illumina TruSeq Stranded *mRNA* Sample Preparation kit. RNAseq was performed on a NovaSeq6000 S4 instrument with a 150 × 150 paired-end format and 100 million read-pair depth. Results were processed with University of Utah Bioinformatics core support. Optical duplicates were removed, adaptors were trimmed, and STAR alignments were generated. Differential gene expression was determined using hciR and DESeq2 (1.30.1) and a 5% false-discovery rate. Pathway analysis was performed using fgsea (1.18.0) using the Hallmarks Molecular Signatures Database. The data presented in this publication were deposited in NCBI’s Gene Expression Omnibus and are accessible through GEO Series accession number GSE232172.

### 4.8. Metabolomics Analysis

Confluent monolayers of BEAS-2B cells grown in T-25 flasks were treated for 0, 4 or 24 h with medium containing 0.2% DMSO or WSPM (20 μg/cm^2^). Treatment medium was aspirated at 0, 4, and 24 h; the cells were rinsed 1× with 5 mL of cold phosphate-buffered saline, and immediately frozen at −80 °C. To each cell flask (*n* = 3; ~2.0 × 10^6^ cells), 450 mL of cold 90% methanol containing the internal standard D_4_-succinic acid (Sigma-Aldrich) was added. The samples were vortexed, incubated at −20 °C for 1 h, and centrifuged at 20,000× *g* for 10 min at 4 °C. Supernatant (400 mL) was then transferred and mixed with D_27_-myristic acid (Sigma-Aldrich). Pooled quality control samples were prepared by removing a fraction of collected supernatant from each sample while process blanks were prepared using only extraction solvent, processed in parallel with the samples. All samples were then dried under vacuum prior to analysis. Samples were analyzed by gas chromatography/mass spectrometry (GC/MS) using an Agilent 7200 GC-QTOF fit with an Agilent 7693A autosampler. Dried samples were suspended in 40 µL of a 40 mg/mL methoxylamine hydrochloride (MOX) (MP Biomedicals, Irvine, CA) in dry pyridine and incubated for 1 h at 37 °C. This solution (25 μL) was added to auto sampler vials to which 60 µL of N-methyl-N-trimethylsilyltrifluoracetamide (MSTFA with 1% TMCS, ThermoFisher) was added via the autosampler and incubated for 30 min at 37 °C. After incubation, samples were vortexed, and 1 µL of the prepared sample was injected into the GC inlet in the split mode with the inlet temperature held at 250 °C. A 5:1 split ratio was used for analysis, and any metabolite that saturated the instrument response at the 5:1 split was re-analyzed at a 50:1 split ratio. The gas chromatograph had an initial temperature of 60 °C for 1 min followed by a 10 °C/min ramp to 325 °C and a hold time of 10 min. A 30 m Agilent Zorbax DB-5MS with 10 m Duraguard capillary column was employed for chromatographic separation. Helium was used as the carrier gas at a rate of 1 mL/min. Data were quantified relative to the internal standard and relative analyte/metabolite peak areas. Processed data were then analyzed for statistical differences and metabolic pathway perturbations using Metaboanalyst 5.0 [75]. Raw data are provided in the Supplementary Materials.

### 4.9. Image Analysis

Images were collected in real time using an Incucyte zoom (Essen Bio Science, Ann Arbor, MI, USA) and converted to movies using an Image Express PICO (Molecular Devices, San Jose, CA, USA). Image files (.jpg or .png) were analyzed using Cellpose 2.0 [76,77] with model training using images collected from multiple experiments. Image masks were created and saved, and then further processed using the Labels to ROIs plugin for Image J [78]. Following a 1 px erosion of the masks, cell area, perimeter, and integrated density were quantified.

### 4.10. Quantification of Known Vasaka Tea Chemicals by LC/MS/MS

Vasicine, vasicinone, isoorientin, isovitexin, and apigenin were quantified using a Thermo Vanquish Flex UPLC System interfaced with an LTQ Velos Pro Linear Ion Trap Mass Spectrometer (ThermoFisher, San Jose, CA, USA). Chromatographic separation of the analytes was achieved using a BEH C_18_ column (150 × 3 mm i.d.; 1.7 μm particle size; Waters, Milford, MA) at 50 °C with gradient elution. The mobile phases were (A) 0.1% formic acid in H_2_O and (B) methanol and a flow rate of 300 mL/minute. The percentage of B varied as follows: 2% B at 0 min, 2%→95% (0 to 7.5 min), hold at 95% (7.5 to 8.5 min), 95%→2% (8.5 to 8.6 min), and hold at 2% from 8.6 to 10 min. A convex gradient (setting = 6 during the 0–7.5 min ramp) was used. The mass spectrometer was operated in positive electrospray ionization, selective reaction-monitoring mode. The MS/MS parameters were optimized by infusion of vasicine into the MS/MS system and the following precursor→product transitions were used: *m/z* 189.1→*m/z* 171.1 for vasicine, *m/z* 203.2→*m/z* 185.1 for vasicinone, *m/z* 449.1→*m/z* 431.1 for isoorientin, *m/z* 433.2→*m/z* 415.1 for isovitexin, and *m/z* 271.1→*m/z* 271.1 + 243.1 for apigenin. Data acquisition and processing were performed using Xcalibur software, and calibration curves were prepared in water using analyte peak area versus concentration.

### 4.11. Discovery Mass Spectrometry

The identification of additional components of Vasaka tea was achieved using positive and negative electrospray LC/MS/MS using a Thermo Vanquish Flex UPLC System interfaced with an LTQ Velos Pro Linear Ion Trap Mass Spectrometer, coupled with dataset interrogation/spectral matching using the Global Natural Products Social (GNPS) Molecular Networking Knowledge Base [38]. Chromatographic fractionation of Vasaka tea was achieved using a BEH C_18_ column (150 × 3 mm i.d.; 1.7 μm particle size; Waters, Milford, MA, USA) at 50 °C with gradient elution. The mobile phases were (A) 0.1% formic acid in H_2_O and (B) methanol and a flow rate of 100 mL/min. The percentage of B varied as follows: 2% B at 0 min, 2%→100% (0 to 50 min), hold at 100% (50 to 55 min), 100%→2% (55.0 to 55.1 min), and hold at 2% (55.1 to 60 min). The mass spectrometer was programmed to assay both positive and negative ions *m/z* 100–750 (in separate acquisitions) using the “double play” data-dependent MS/MS mode. MS/MS scans were triggered by analytes having a signal intensity set at ~5× baseline using a collision energy of 30% or 45% (separate acquisitions). The top four peaks of each scan were analyzed, and dynamic exclusion was active. The parameters were as follows: repeat count = 3; repeat duration = 5 s; exclusion list size = 25; and exclusion duration = 5 s. Data were analyzed using GNPS. The .raw files were converted to mzML files using the GNPS quickstart feature. The files were then uploaded into GNPS and processed using the Library Search feature. Default criteria were used with the exception that the minimum matched peaks = 4 and top hits per spectrum = 5.

### 4.12. Quantification of Oxylipins in Vasaka Tea by LC/MS/MS

Quantification of 9(10)- and 12(13)-EpOME; 9(10)- and 12(13)-DiHOME, 9(S)- and 13(S)-HODE, and 9- and 13-oxoODE was achieved using a Thermo Vanquish Flex UPLC System interfaced with an LTQ Velos Pro Linear Ion Trap Mass Spectrometer. Chromatographic separation was achieved using a BEH C_18_ column (150 × 3 mm i.d.; 1.7 μm particle size; Waters, Milford, MA) at 35 °C with gradient elution. The mobile phases were (A) 0.1% acetic acid in H_2_O and (B) 9:1 acetonitrile/isopropanol, and a flow rate of 250 mL/minute was used. The percentage of B varied as follows: 52.5% B at 0 min, 52.5%→57.5% (0 to 6 min), 62.5% at 6.1 min, 62.5%→77% (6.1 to 12.5 min), 77%→95% (12.5 to 16 min), 95% hold (16 to 19 min), 95%→5.25% (19 to 19.1 min), and 52.5% hold until 22 min. Negative electrospray ionization selective reaction monitoring was used. The MS/MS parameters were optimized by infusion of lipid standard (Cayman Chemical) solutions into the MS/MS system. The precursor→product transitions used were as follows: *m/z* 313.2→*m/z* 194.0 + 183.1 for 12(13)-DiHOME, *m/z* 313.2→*m/z* 201.1 + 171.1 for 9(10)-DiHOME, *m/z* 295.3→*m/z* 195.1 + 171.1 for 13(S) and 9(S)-HODE, as well as 12(13)- and 9(10)-EpOME, and *m/z* 293.2→*m/z* 185.1 + 113.1 for 13- and 9-oxoODE. The precursor-to-product ion transitions used for the internal standards were as follows: *m/z* 317.2→*m/z* 185.1 for 9(10)-EpOME-d_4_, *m/z* 317.2→*m/z* 203.1 for 12(13)-EpOME-d_4_, *m/z* 299.2→*m/z* 172.0 for 9(10)-DiHOME-d_4_, and *m/z* 299.2→*m/z* 198.0 for 12(13)-DiHOME-d_4_. Data acquisition and processing were performed using Xcalibur software with calibration curves prepared in water using the ratio of analyte to internal standard peak.

### 4.13. Statistical Analysis

Graphing and statistical analyses were performed using GraphPad Prism 9.5.0 software. Values are represented as the mean ± SD unless stated otherwise. For comparisons between two groups, the unpaired *t*-test was used. One- or two-way ANOVA with Tukey’s or a Bonferroni post-test was used for multiple comparisons. A *p*-value <0.05 was considered significant.

## 5. Conclusions

A tea prepared from a Vasaka supplement was found to inhibit the TRPA1 receptor and attenuate WSPM-induced AEC damage and ectopic *MUC5AC* expression in vitro. These effects were attributable to an attenuation of TRPA1-induced ER stress and, to a lesser extent, oxidative stress by selected flavones (i.e., apigenin) and linoleic-derived oxylipins (i.e., 9,10-EpOME) present in the tea. It is possible that these mechanisms may underlie the purported beneficial effects of Vasaka and Vasaka tea in treating respiratory malaise, and that a standardized Vasaka tea/supplement or pure apigenin and/or 9,10-EpOME may provide therapeutic benefit.

## Data Availability

All data presented in the study are available in the main manuscript or Supplementary Materials. The RNA sequencing data presented in this publication were deposited in the National Center for Biotechnology Information (NCBI) Gene Expression Omnibus (GEO) and are accessible through GEO Series accession number GSE232172. Raw data will be made available upon request.

## Supplementary Materials

The following supporting information can be downloaded at https://www.mdpi.com/article/10.3390/ph16060890/s1: Figure S1A–E. comparison of the expression of leading-edge genes associated with the (A) hypoxia, (B) xenobiotic metabolism, (C) Kras up and down, (D) glycolysis and cholesterol homeostasis, and (E) mitotic spindle hallmark pathways obtained from RNA sequencing analysis of HBEC3-KT cells treated with WSPM and Vasaka tea; Figure S2. Comparison of metabolomic pathway signatures in control and WSPM-treated BEAS-2B cells, as generated by data analysis using Metaboanalyst 5.0; Figure S3. Movies of HBEC3-KT cells treated with vehicle, WSPM, or WSPM + Vasaka tea; Figure S4A,B. LC/MS/MS selective reaction monitoring chromatogram and mirror match spectra for vasicine, vasicinone, isoorientin, isovitexin, and apigenin analysis; Figure S5a,b. +ESI and −ESI LC/MS chromatograms from untargeted LC/MS/MS analysis of Vasaka tea; Figure S6A–F. Mirror match MS/MS spectra for the identification of oxylipins in Vasaka tea and LC/MS/MS selective reaction monitoring chromatogram from oxylipin quantification by LC/MS/MS; Figure S7. Data showing the activation and inhibition of TRPA1 activity by selected oxylipins.
