# Supplementary material for: Inhibition of TRPA1, Endoplasmic Reticulum Stress, Human Airway Epithelial Cell Damage, and Ectopic MUC5AC Expression by Vasaka (Adhatoda vasica; Malabar Nut) Tea
Source: Pharmaceuticals (Basel). 2023 Jun 17;16(6):890. doi: 10.3390/ph16060890 (PMC10303053; doi:10.3390/ph16060890)

Supplemental Figure S1A

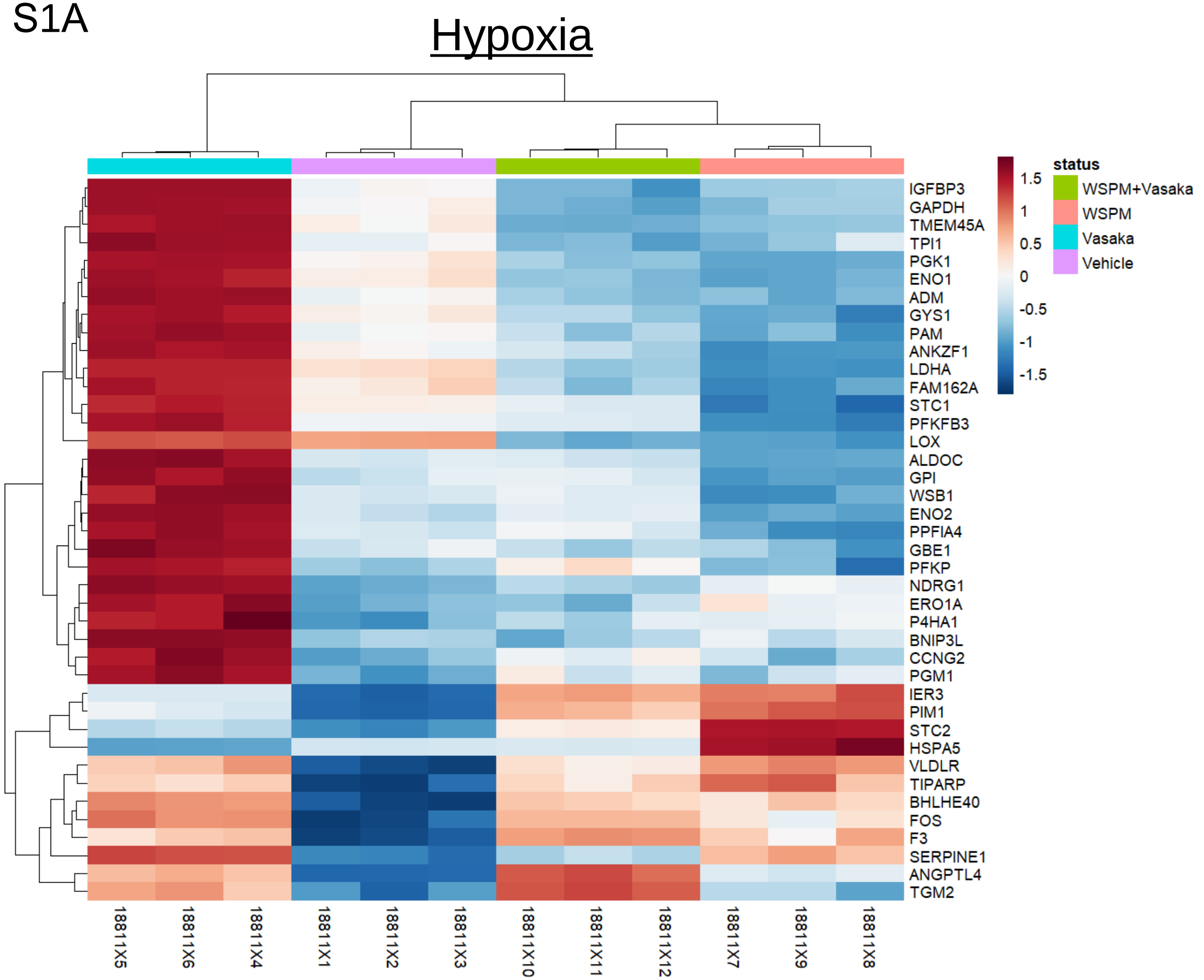

Supplemental Figure S1B

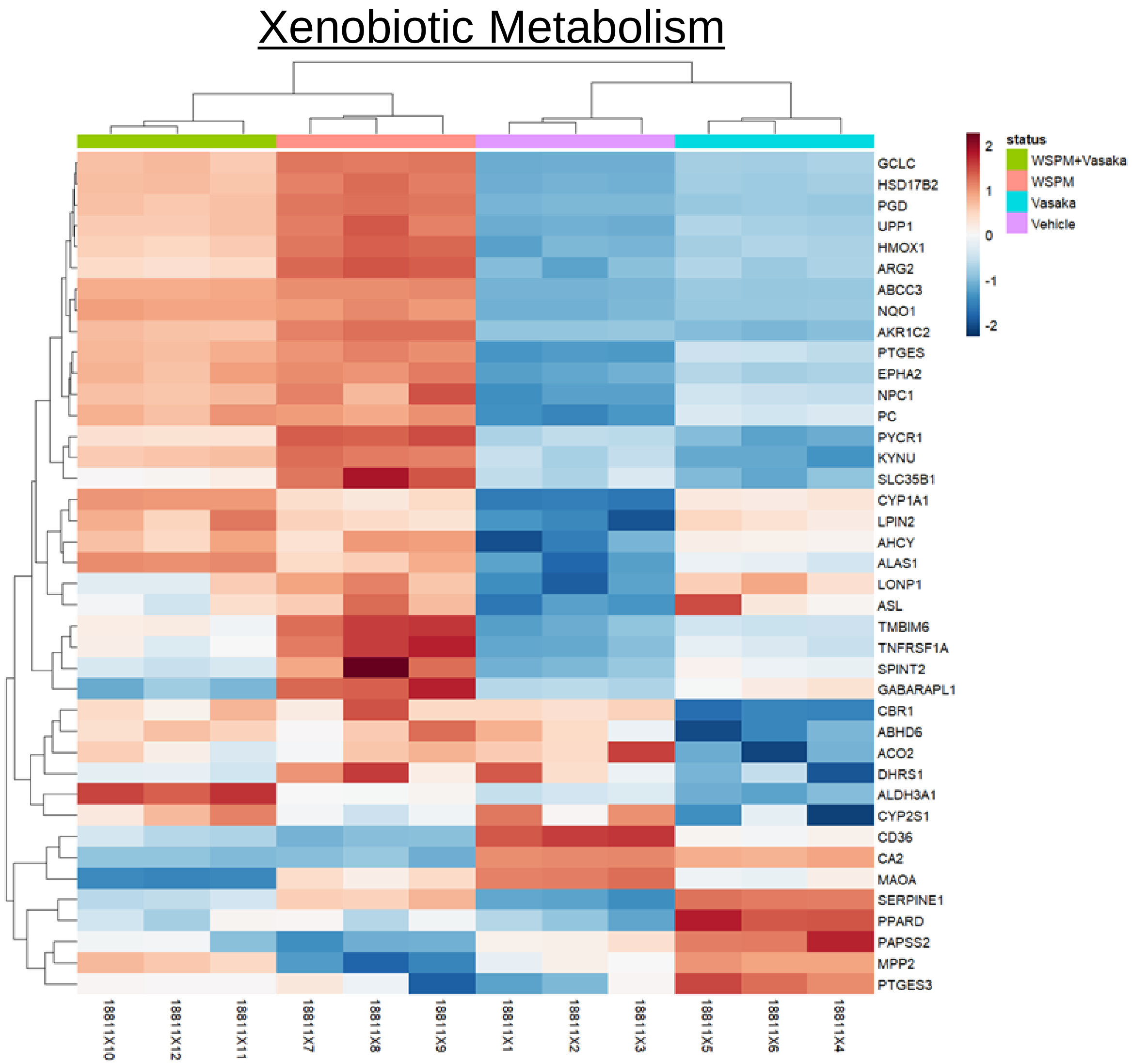

Supplemental Figure S1C

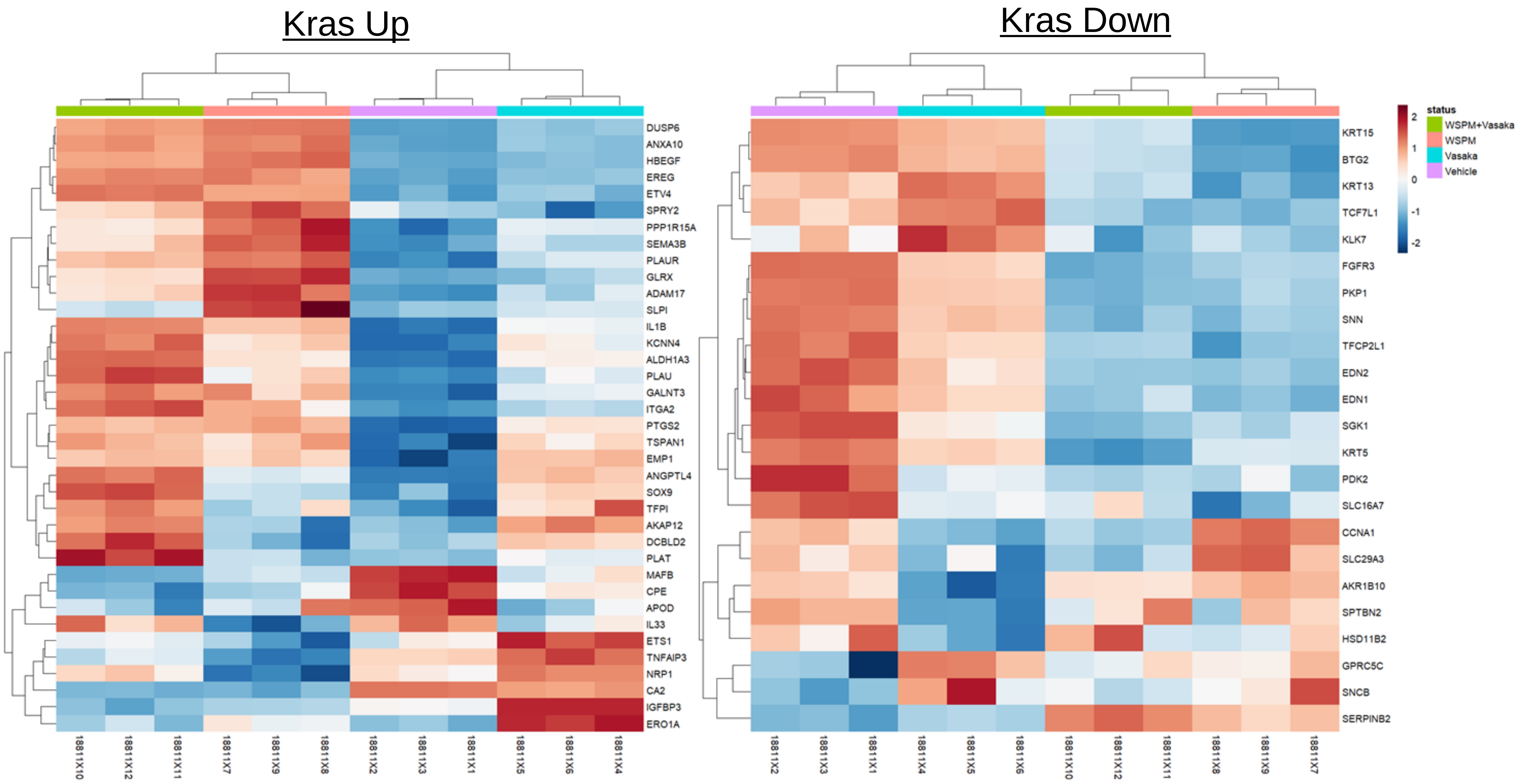

Supplemental Figure S1D

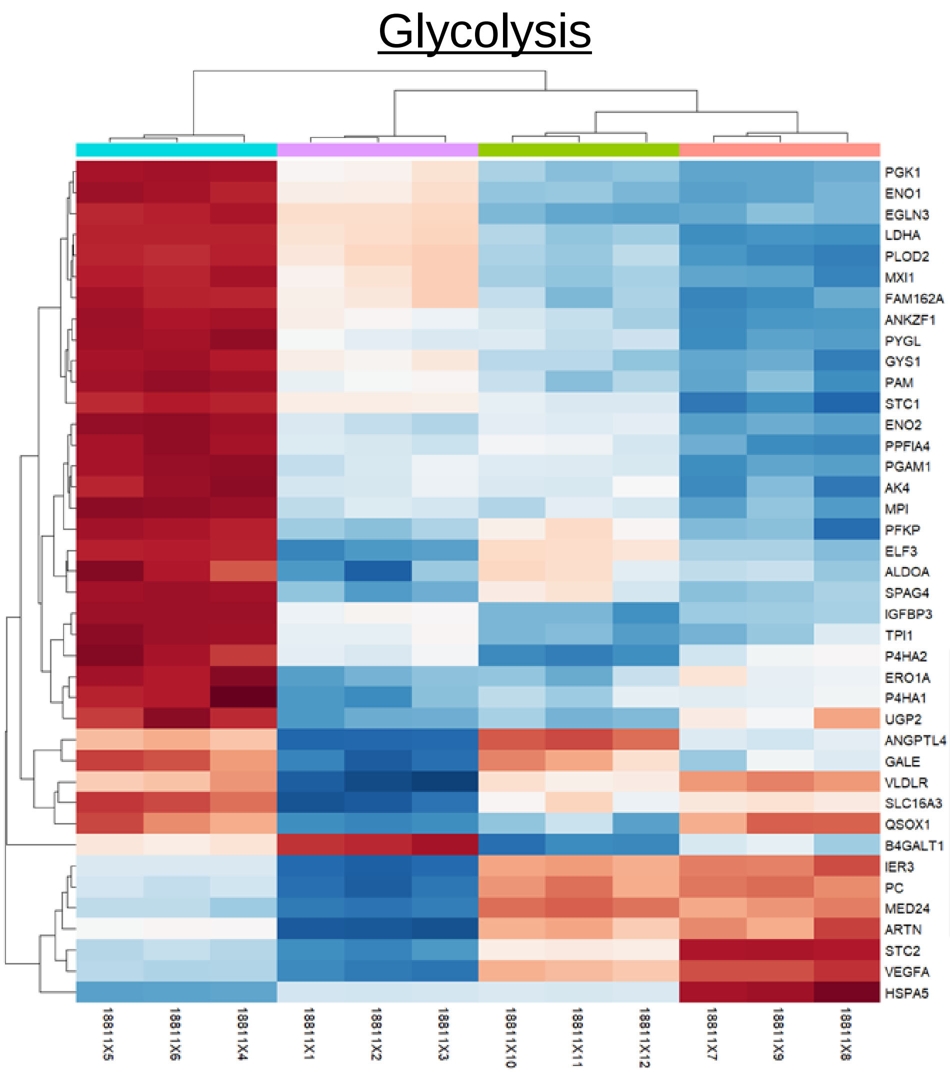


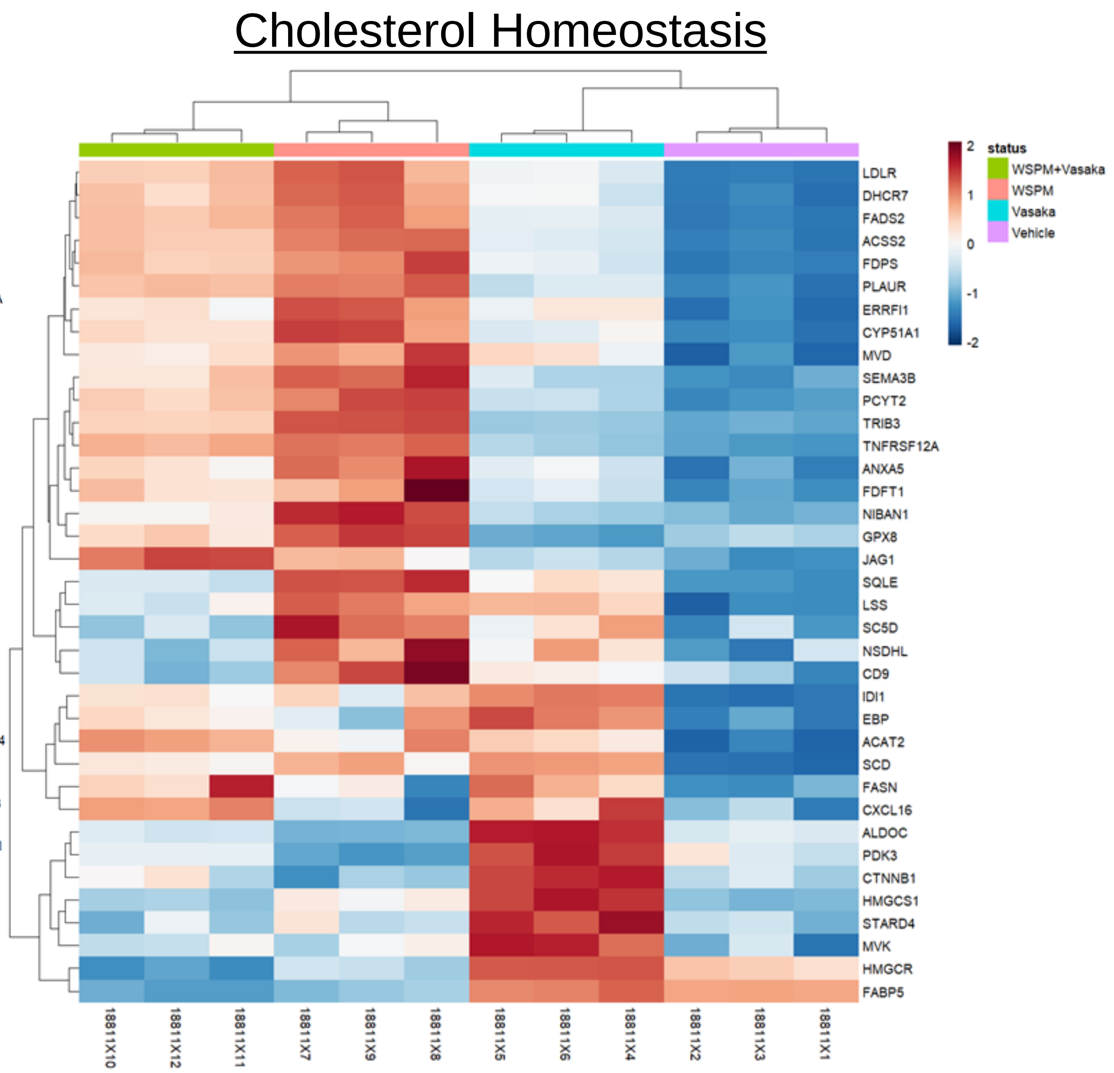

Supplemental Figure S1E

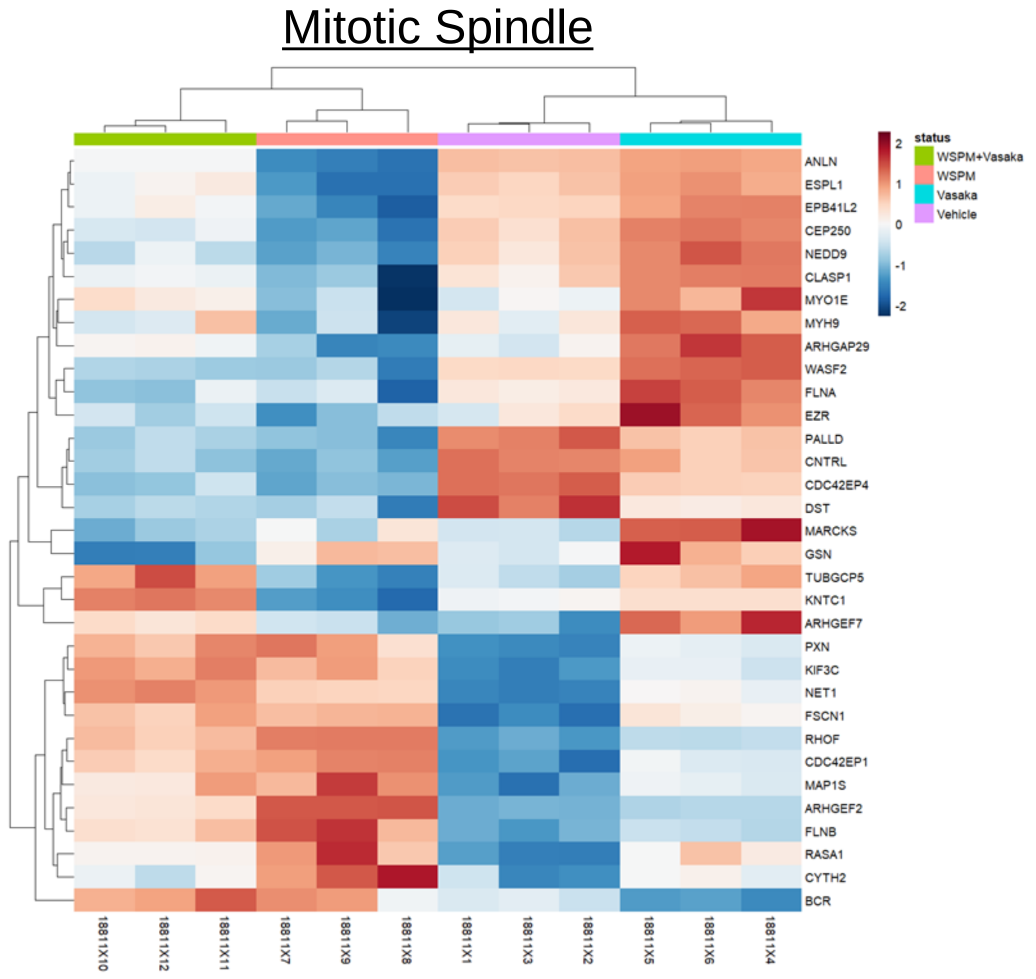

# Supplemental Figure S2A

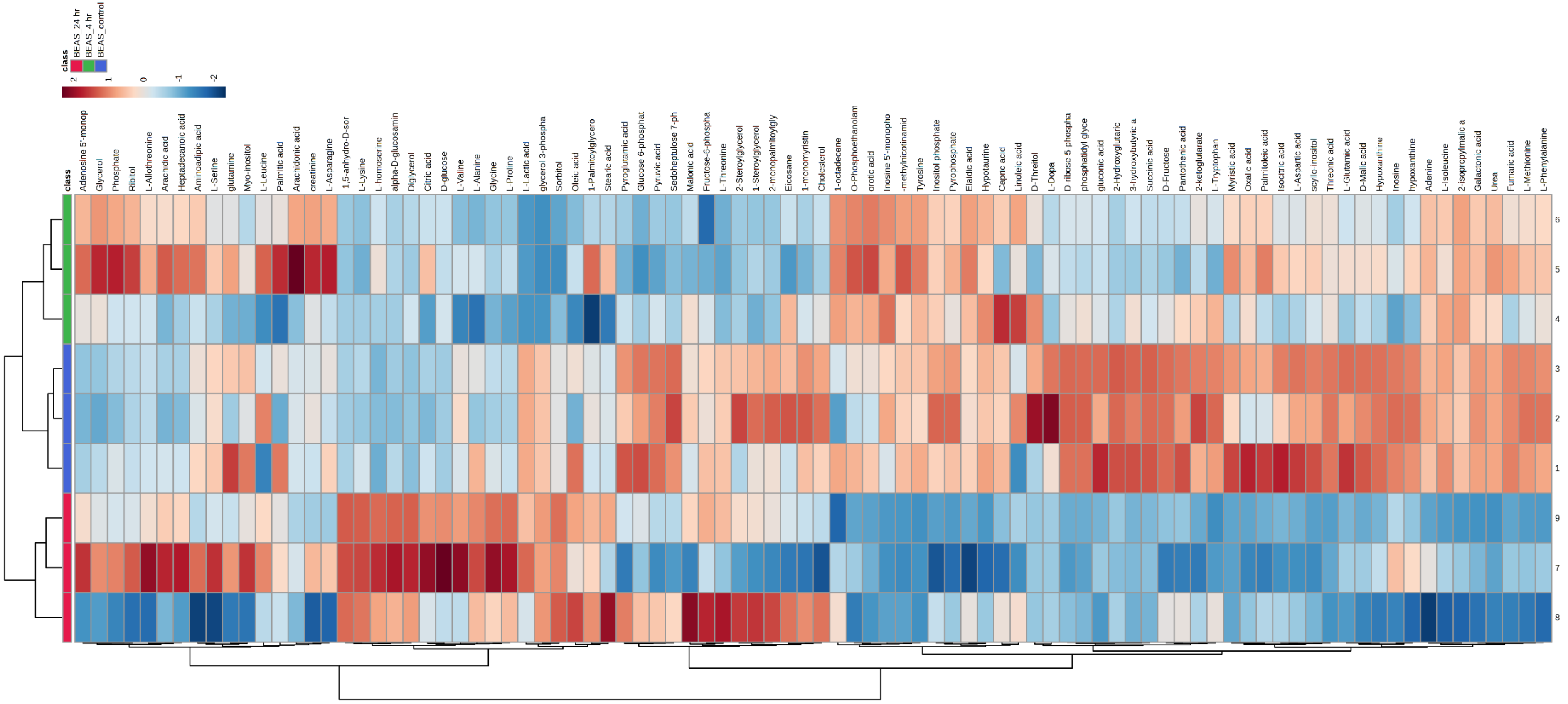

# Supplemental Figure S2B

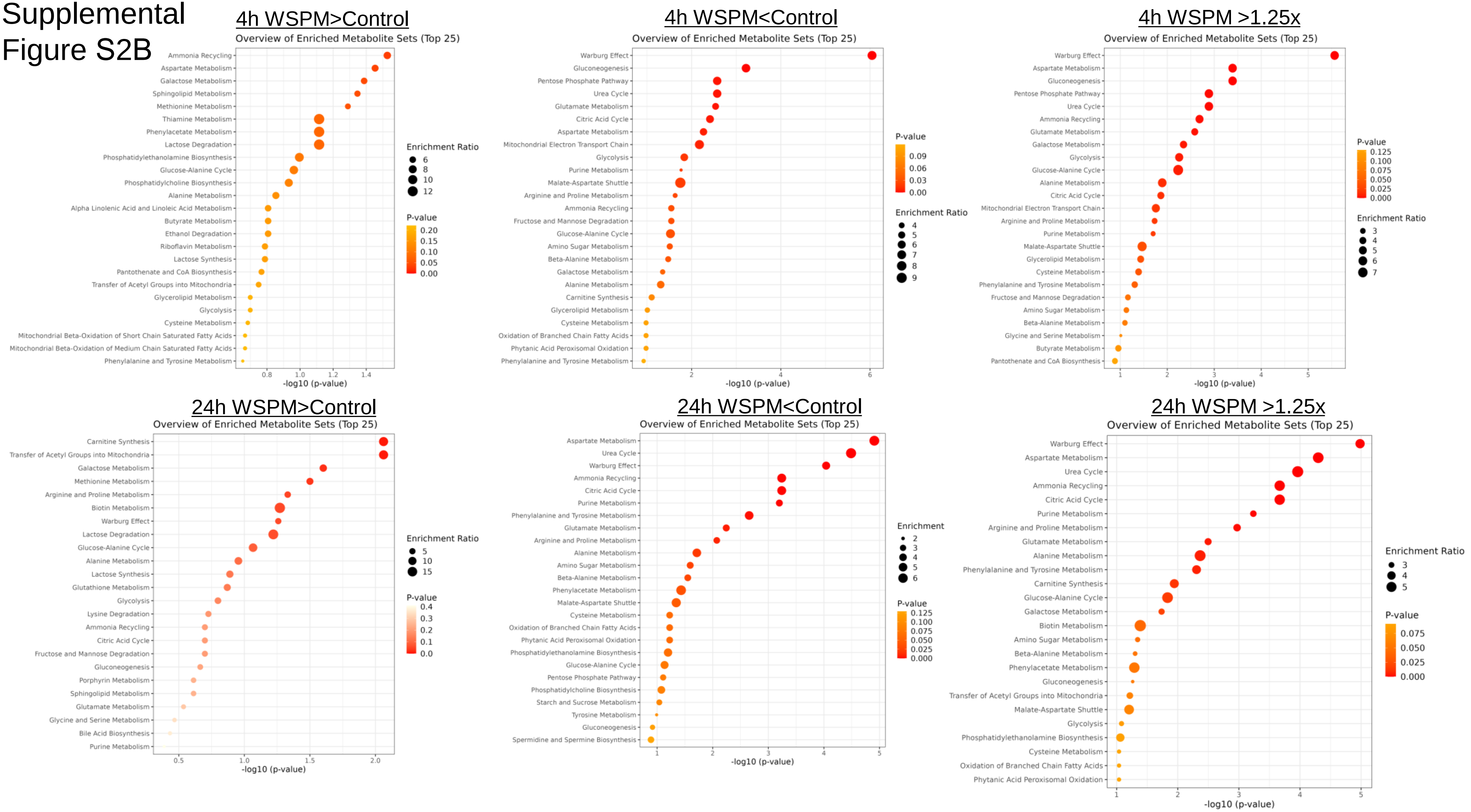

Supplemental Figure S3 (Movies)

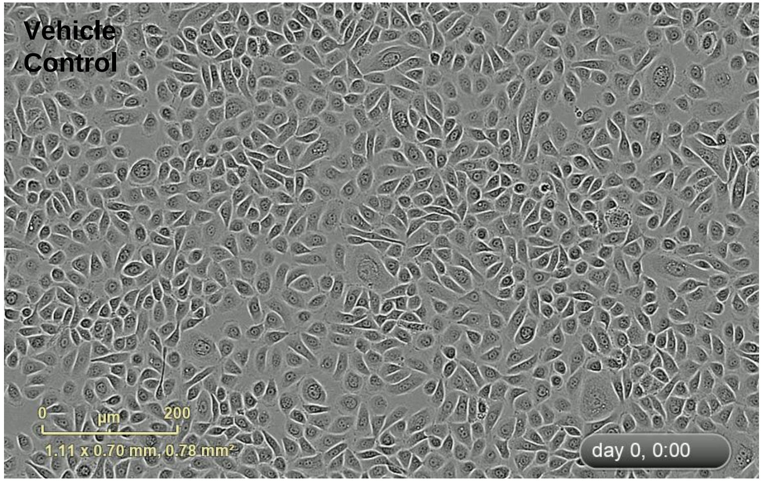


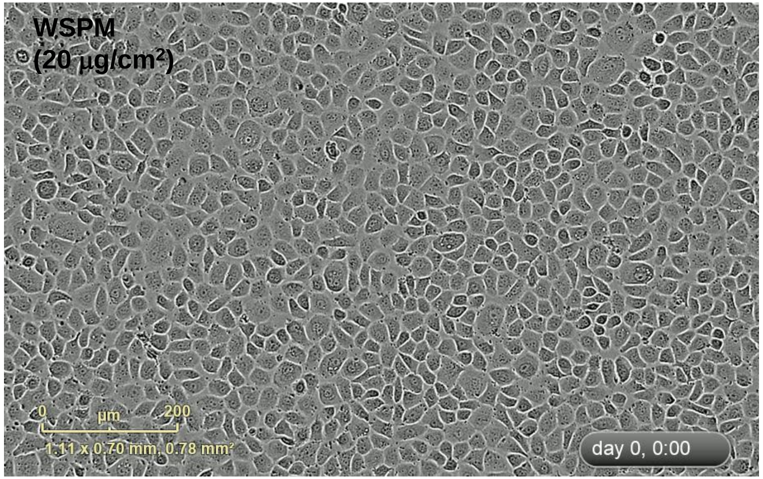


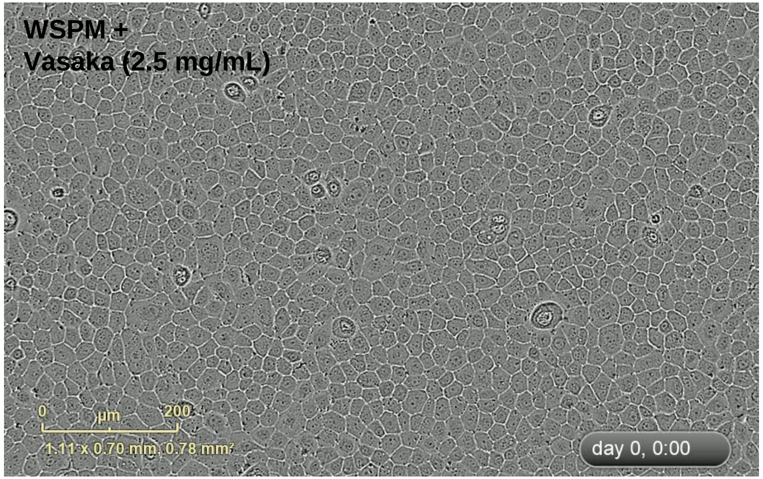

# Supplemental Figure S4A

## Vasaka tea vs. Standard mix

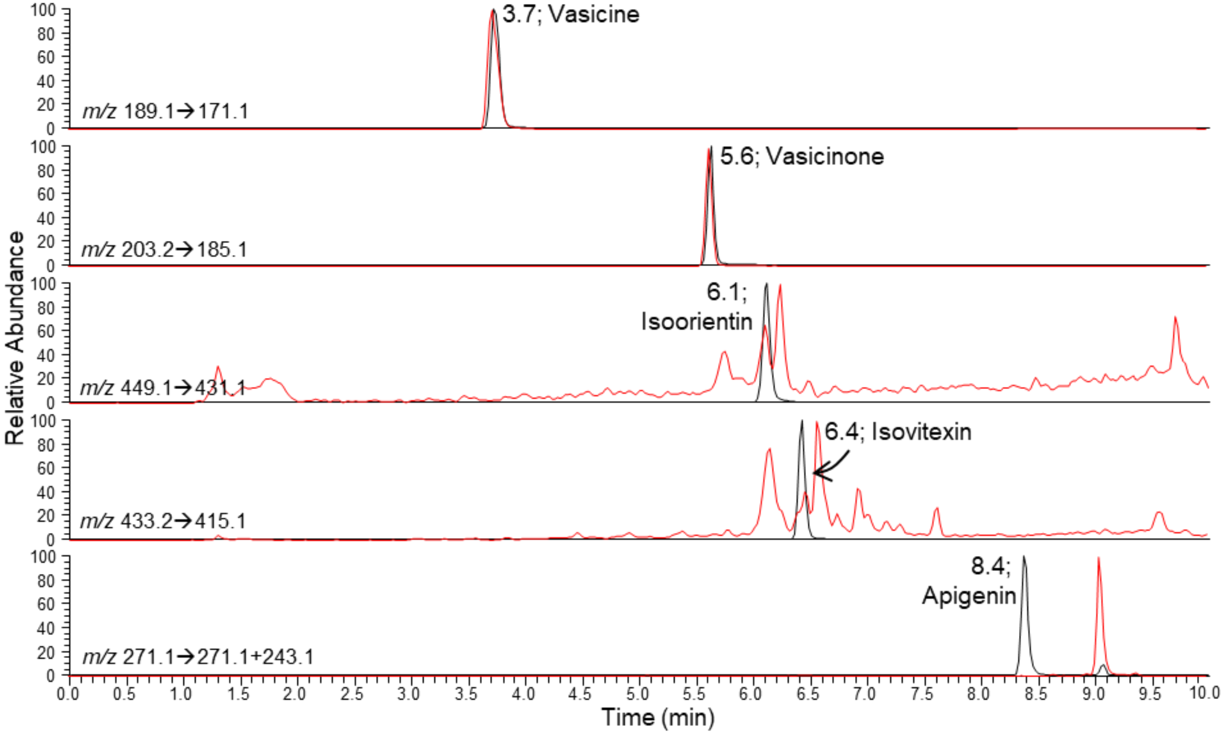

# Supplemental Figure S4B

## Vasicine (peganine) in Vasaka

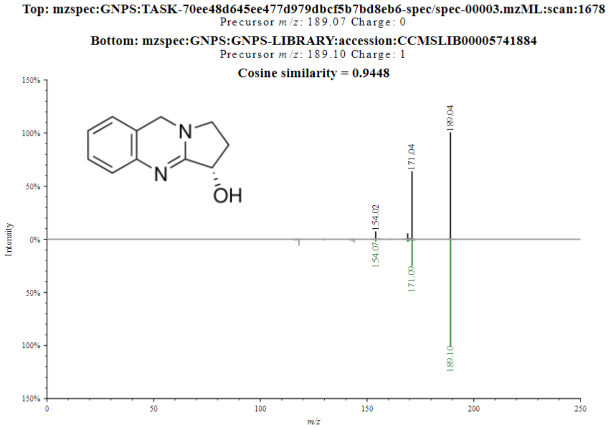


## Isoorientin (Homoorientin; Luteolin 6-C-glucoside) in Vasaka

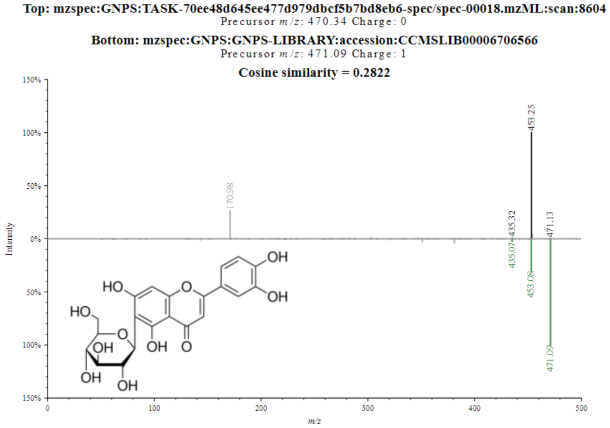


## Vasicinone in Vasaka

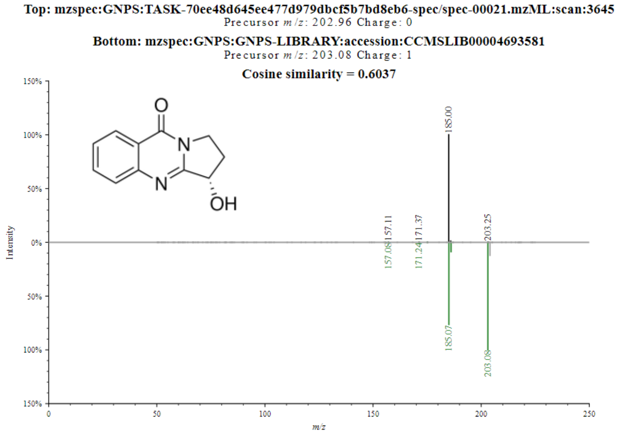


## Isovitexin (Homovitexin; Apigenin 6-C-glucoside) in Vasaka

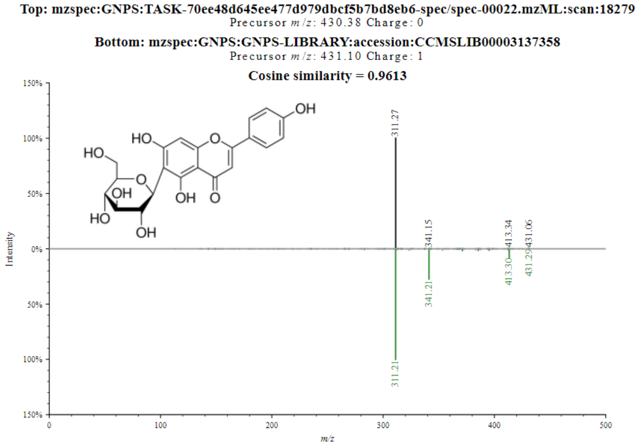

Supplemental Figure S5a

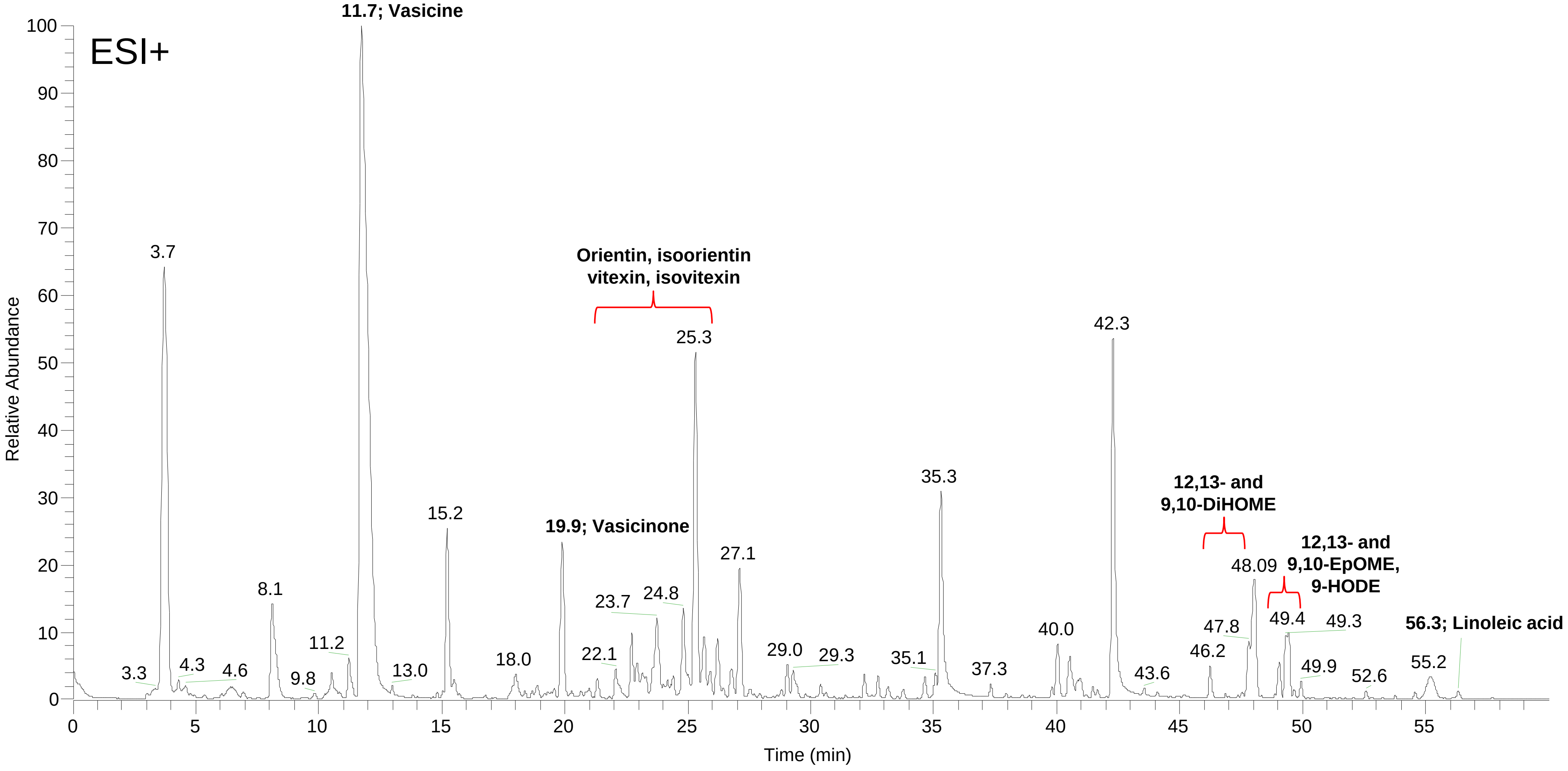

Supplemental Figure S5b

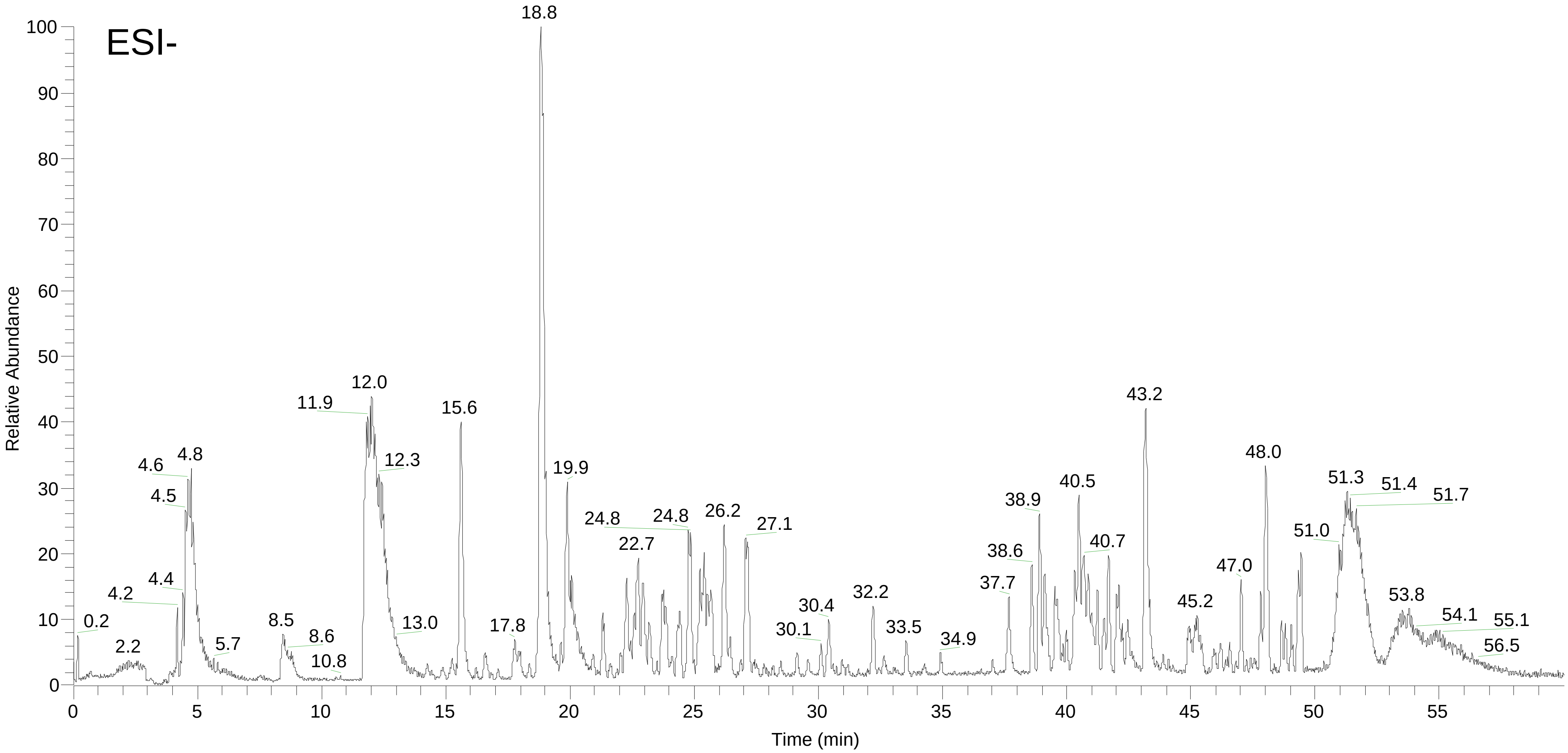

# Supplemental Figure S6A

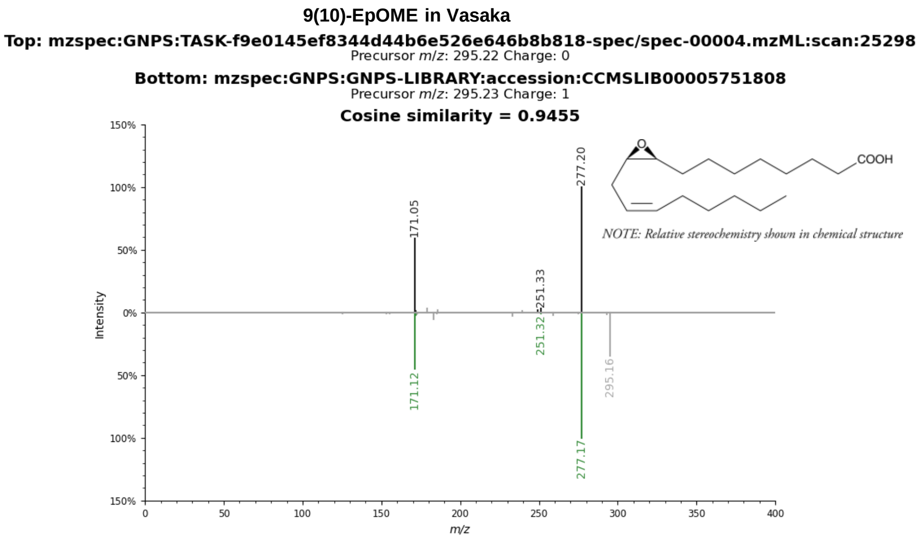


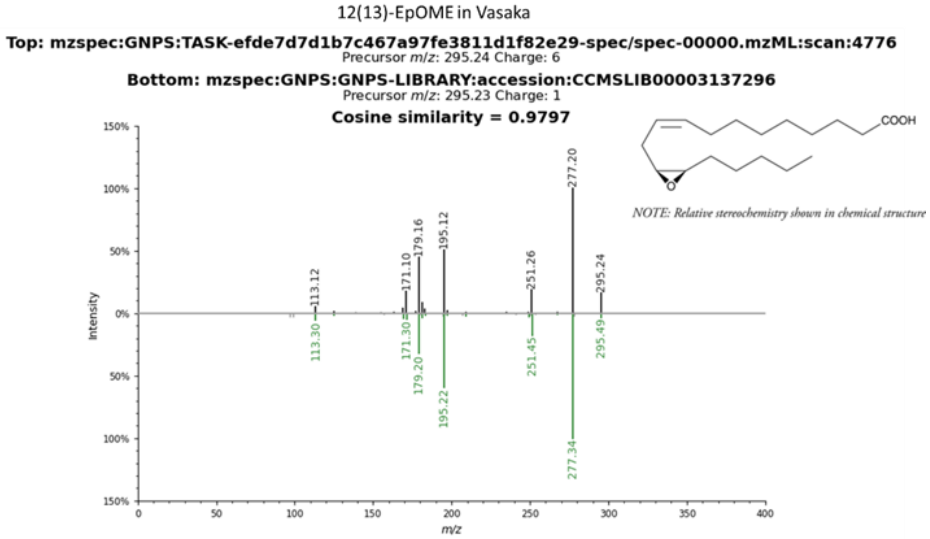

# Supplemental Figure S6B

9,10-DiHOME in Vasaka

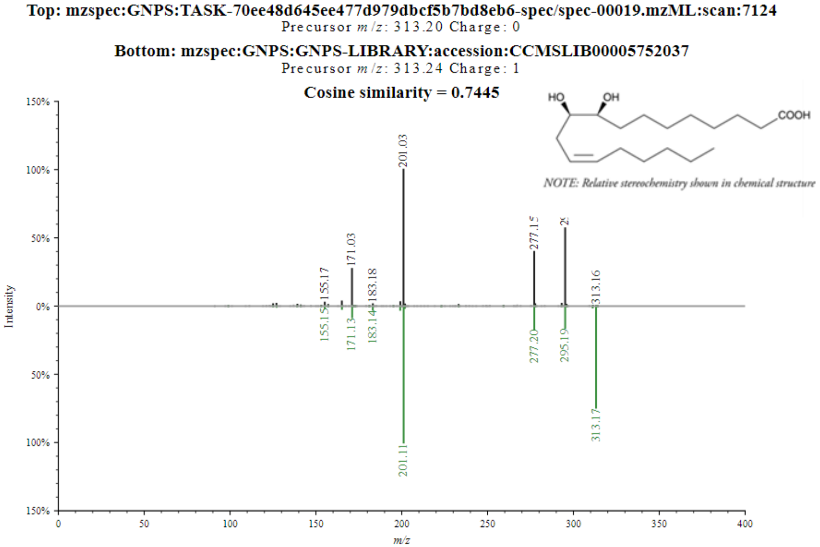


12,13-DiHOME in Vasaka

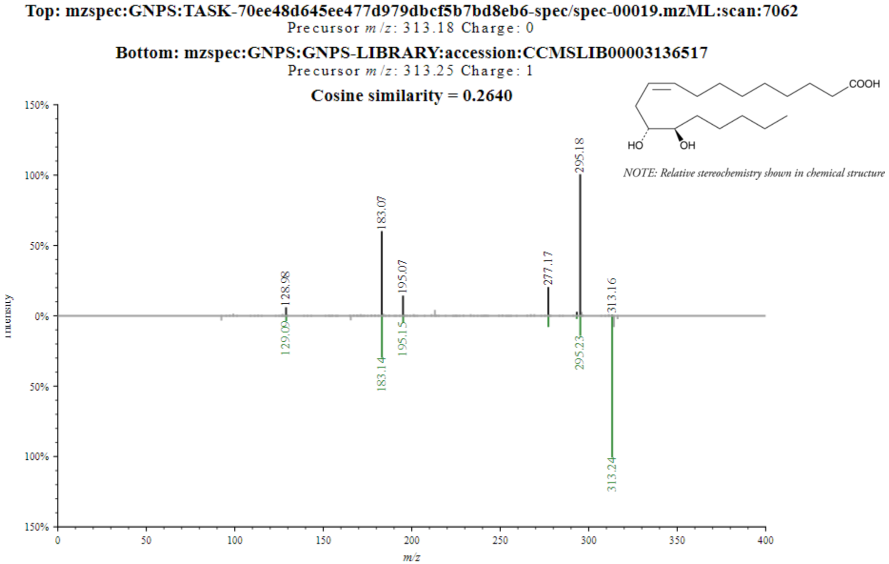

# Supplemental Figure S6C

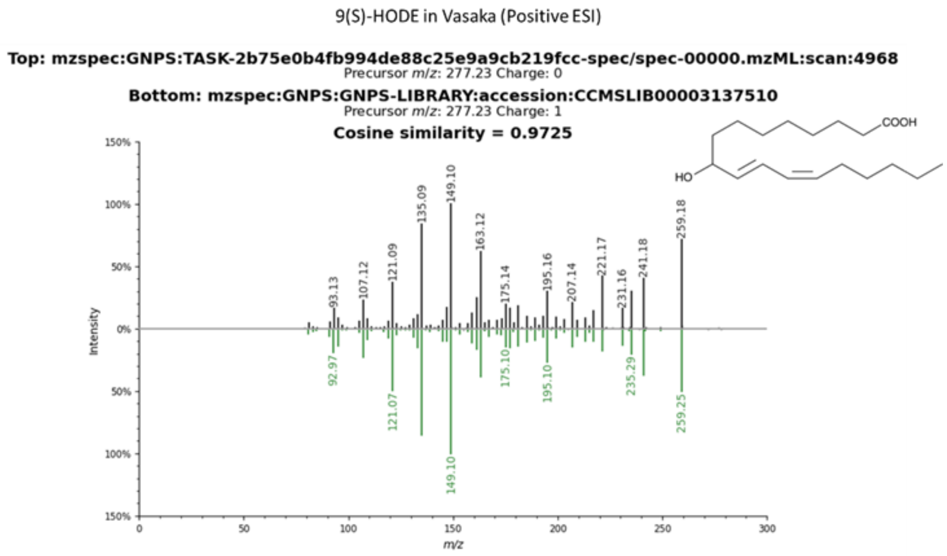


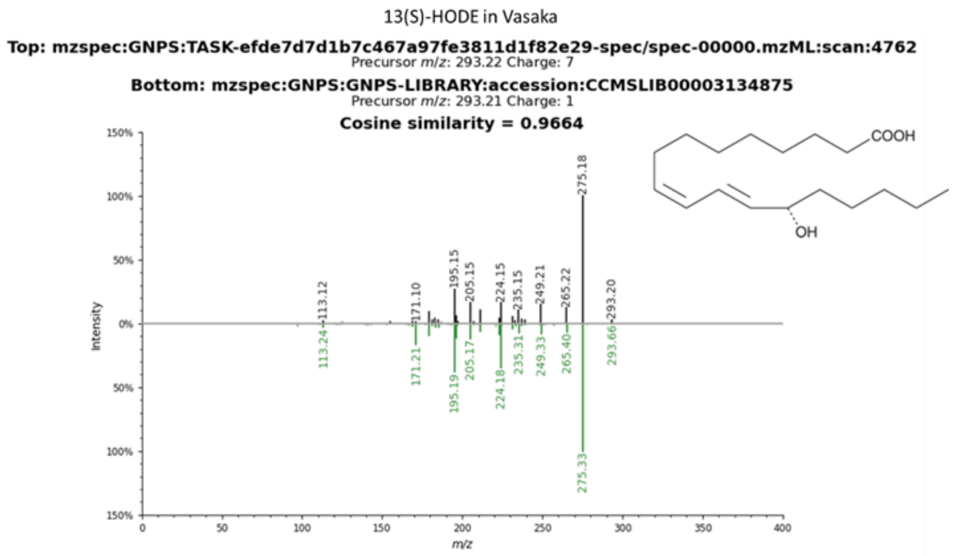

# Supplemental Figure S6D

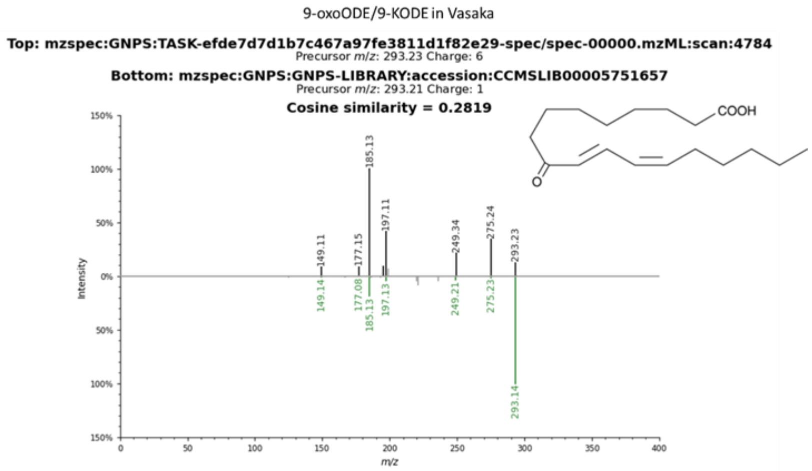

# Supplemental Figure S6E

## 9-HpODE in Vasaka

**Top: mzspec:GNPS:TASK-70ee48d645ee477d979dbcf5b7bd8eb6-spec/spec-00019.mzML:scan:6594**
Precursor *m/z*: 311.16 Charge: 0

**Bottom: mzspec:GNPS:GNPS-LIBRARY:accession:CCMSLIB00005752901**
Precursor *m/z*: 311.22 Charge: 1

**Cosine similarity = 0.9226**

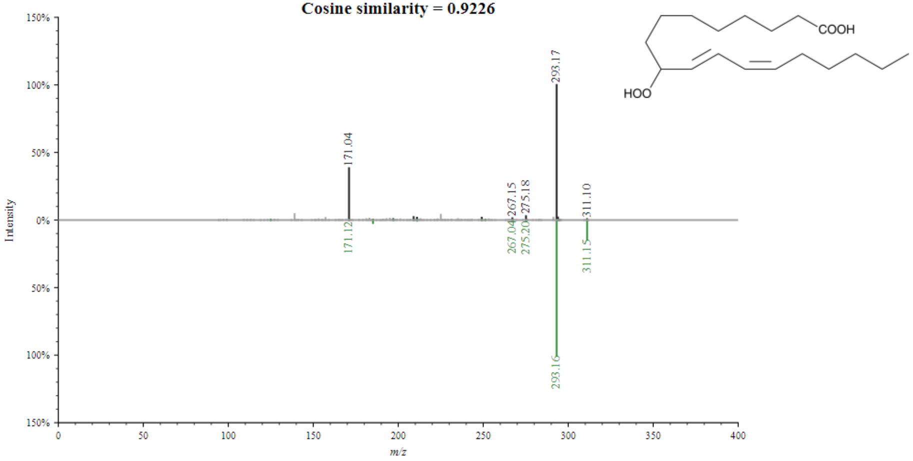


## 13-HpODE in Vasaka

**Top: mzspec:GNPS:TASK-70ee48d645ee477d979dbcf5b7bd8eb6-spec/spec-00019.mzML:scan:7150**
Precursor *m/z*: 311.15 Charge: 0

**Bottom: mzspec:GNPS:GNPS-LIBRARY:accession:CCMSLIB00005751861**
Precursor *m/z*: 311.22 Charge: 1

**Cosine similarity = 0.9023**

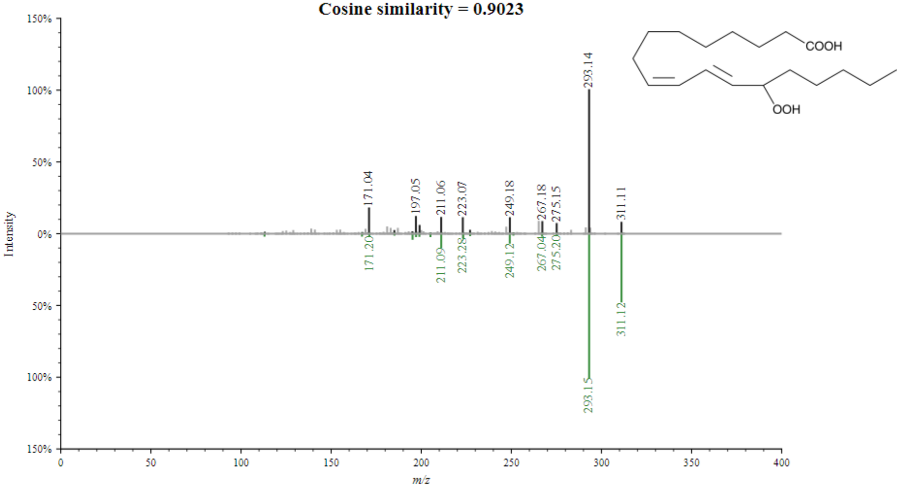

Supplemental Figure S6F

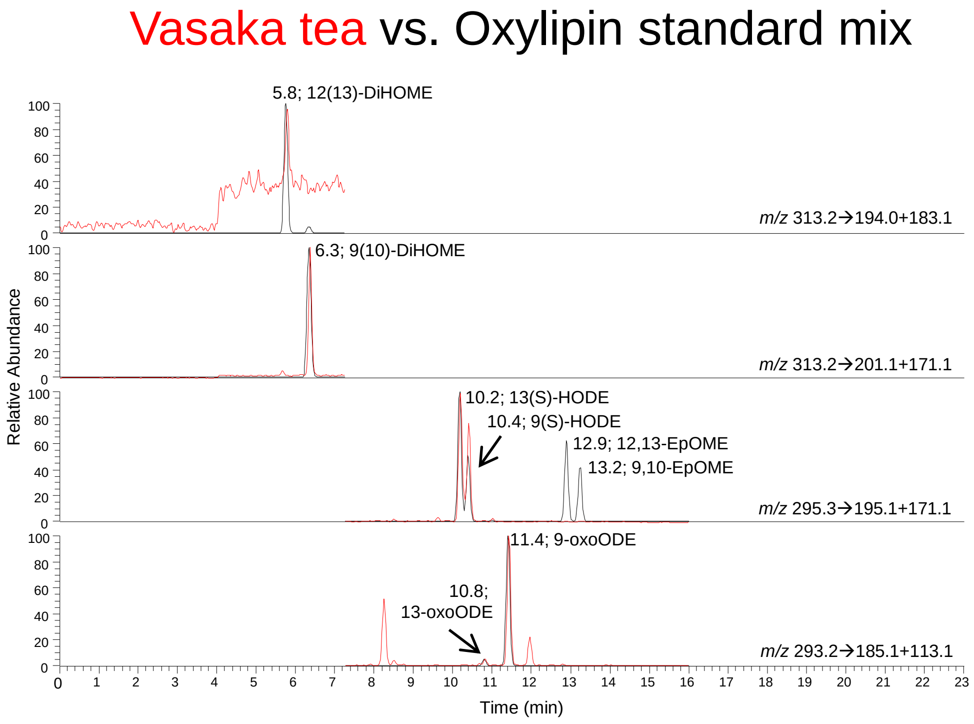

# Supplemental Figure S7

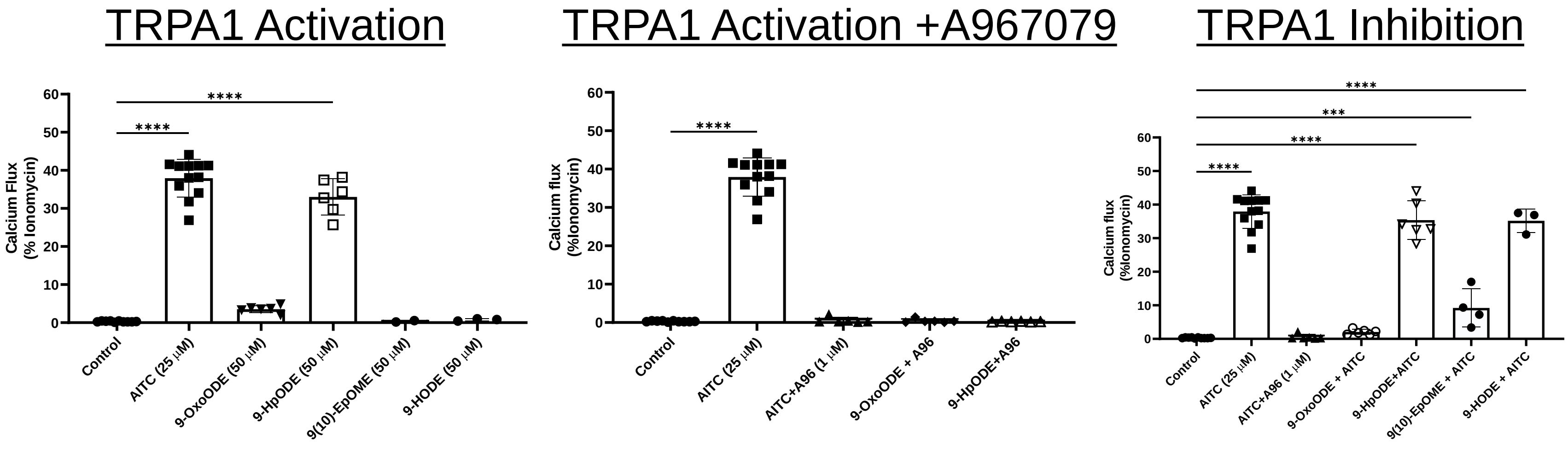

Supplement: Supplementary file 1 [file pharmaceuticals-16-00890-s001.zip › Vasaka Paper Supplemental Figures.pdf]
